# A novel compound heterozygous leptin receptor mutation causes more severe obesity than in *Lepr*^*db/db*^ mice

**DOI:** 10.1016/j.jlr.2021.100105

**Published:** 2021-08-11

**Authors:** Claudia Berger, Henrike O. Heyne, Tina Heiland, Sebastian Dommel, Corinna Höfling, Esther Guiu-Jurado, Jana Lorenz, Steffen Roßner, Michael Dannemann, Janet Kelso, Peter Kovacs, Matthias Blüher, Nora Klöting

**Affiliations:** 1 Medical Department III, Endocrinology, Nephrology, Rheumatology, CRC1052, University of Leipzig Medical Center, Leipzig, Germany; 2 Medical Department, Institute for Human Genetics, University of Leipzig Medical Center, Leipzig, Germany; 3 Institute for Molecular Medicine Finland: FIMM, Helsinki, Finland; 4 Broad Institute of Harvard and MIT, Cambridge, MA, USA; 5 Medical Department, Neurology and Dermatology, Division of Gastroenterology and Rheumatology, University of Leipzig Medical Center, Leipzig, Germany; 6 Paul-Flechsig-Institute for Brain Research, University of Leipzig, Leipzig, Germany; 7 Department of Evolutionary Genetics, Max Planck Institute for Evolutionary Anthropology, Leipzig, Germany; 8 Institute of Genomics, University of Tartu, Tartu, Estonia; 9 Helmholtz Institute for Metabolic, Obesity and Vascular Research (HI-MAG) of the Helmholtz Zentrum München at the University of Leipzig, Leipzig, Germany

**Keywords:** *Lepr*, leptin receptor mutation, obesity, compound heterozygous, genetic background, ADCY3, adenylate cyclase 3, AT, adipose tissue, BC, backcross, DM, dorsomedial hypothalamic nucleus, epi, epigonadal, Jak, Janus kinase, Lepr, leptin receptor, LH, lateral hypothalamic area, Mapk, mitogen-activated protein kinase, PVN, paraventricular hypothalamic nucleus, QTL, quantitative trait locus, sc, subcutaneous, Stat, signal transducers and activators of transcription, VMN, ventromedial hypothalamus

## Abstract

The leptin receptor (*Lepr*) pathway is important for food intake regulation, energy expenditure, and body weight. Mutations in leptin and the *Lepr* have been shown to cause early-onset severe obesity in mice and humans. In studies with C57BL/6NCrl mice, we found a mouse with extreme obesity. To identify a putative spontaneous new form of monogenic obesity, we performed backcross studies with this mouse followed by a quantitative trait locus (QTL) analysis and sequencing of the selected chromosomal QTL region. We thereby identified a novel *Lepr* mutation (C57BL/6N-*Lepr*^L536Hfs*^^6-1NKB^), which is located at chromosome 4, exon 11 within the CRH2-leptin-binding site. Compared with C57BL/6N mice, *Lepr*^L536Hfs*^^6^ develop early onset obesity and their body weight exceeds that of *Lepr*^*db/db*^ mice at an age of 30 weeks. Similar to *Lepr*^*db/db*^ mice, the *Lepr*^L536Hfs*^^6^ model is characterized by hyperphagia, obesity, lower energy expenditure and activity, hyperglycemia, and hyperinsulinemia compared with C57BL/6N mice. Crossing *Lepr*^*db/wt*^ with *Lepr*^L536Hfs*^^6/wt^ mice results in compound heterozygous *Lepr*^L536Hfs*^^6/*db*^ mice, which develop even higher body weight and fat mass than both homozygous *Lepr*^*db/db*^ and *Lepr*^L536Hfs*^^6^ mice. Compound heterozygous Lepr deficiency affecting functionally different regions of the Lepr causes more severe obesity than the parental homozygous mutations.

The discovery of leptin has changed our understanding of how circuits in the central nervous system that modulate energy intake and expenditure are regulated in response to changes in body energy stores (1, 2). Secreted from adipose tissue (AT), leptin binds to leptin receptor (Lepr), a type I cytokine receptor, expressed in various tissues including neurons of the hypothalamus. Leptin pathway not only plays a key role in regulating appetite and food intake, energy expenditure, but also in fertility and glucose homeostasis. *Leptin* or the *obese* (*ob*) gene was discovered in 1994 as a molecule that caused severe obesity in *ob/ob* mice (3). Leptin plasma concentrations correlate with AT mass and decrease after weight loss (3, 4). Loss-of-function mutations in the *leptin* gene cause obesity in *ob/ob* mice (5, 6) and also, rare cases of human obesity with severe early onset and hyperphagia (7, 8).

Comparable to leptin deficient *ob/ob* mice, mutations in the *Lepr* cause obesity in *Lepr*^*db/db*^ mice (9, 10) and other models (11). Moreover, mutations in the human *LEPR* cause obesity and pituitary dysfunction (12). In addition, *Lepr*^*db/db*^ mice develop hyperglycemia, hyperphagia, fatty liver, insulin resistance, and are infertile (13, 14).

Lepr exists in five different isoforms due to alternative splicing and enzymatic shedding in mice (10, 15, 16). The Lep-Ra, -Rb, -Rc, and -Rd isoforms share a common extracellular and transmembrane domain while the intracellular part differs (7). The long Lep-Rb represents the active isoform for signal transduction and is highly expressed in nuclei of hypothalamus (9, 10, 17, 18, 19). Lep-Ra, -Rc, and -Rd vary in intracellular length and are expressed in peripheral tissues such as the liver, lung, heart, testis, and AT (17, 18). The shortest isoform, Lep-Re, only consists of the extracellular domain and is secreted into the blood as soluble Lepr (20).

Lepr signaling is activated by leptin and results in Janus kinase (Jak)/signal transducers and activators of transcription (Stat) signaling (1, 10). While the stoichiometry of the leptin-Lepr-complex is still under discussion, Jak2 autophosphorylation is initiated by conformational changes within the leptin-Lepr-complex, followed by phosphorylation of Lepr tyrosine residues Y985, Y1077, and Y1138 (21, 22, 23, 24). Phosphorylated Y985 recruits two proteins, which activate the mitogen-activated protein kinase (Mapk) pathway (1). Stat3 is recruited by phosphorylated Y1138. Mice lacking Stat3 in Lepr-expressing neurons develop hyperphagic obesity supporting the important role of Stat3 signaling in mediating central leptin effects (25, 26).

Here, we describe an extremely obese mouse phenotype among offspring from backcross studies with C57BL/6NCrl mice and the discovery of a novel spontaneous single nucleotide deletion in the *Lepr* named C57BL/6N-*Lepr*^L536Hfs*^^6-1NKB^ (*Lepr*^L536Hfs*^^6^) (27). To further explore the impact of this novel *Lepr* mutation, we compared the strain with the well-established *Lepr*^*db/db*^ mouse and generated a compound heterozygous mouse model of *Lepr*^L536Hfs*^^6*/db*^ mice.

## Materials and methods

### Mice and breeding

All mice were housed in pathogen-free facilities in groups of three to four at 22 ± 2°C on a 12 h light/dark cycle. Animals were kept in individually ventilated cages under specific pathogen-free conditions at the University of Leipzig. All animals had free access to water and were fed with standard chow diet (Sniff GmbH, Soest, Germany). The C57BL/6N-*Lepr*^L536Hfs*^^6-1NKB^ (*Lepr*^L536Hfs*^^6^) and *Lepr*^*db/db*^ (Janvier Labs, France) mice were backcrossed onto C57BL/6NTac background for at least six generations by heterozygous breeding. C57BL/6NTac littermates served as wildtype (wt) controls. Compound heterozygous mice (*Lepr*^L536Hfs*^^6/*db*^) were generated by heterozygous crossing heterozygous *Lepr*^L536Hfs*^^6/wt^ with *Lepr*^*db/wt*^ mice.

### QTL analysis

Two lean brothers of the firstly identified female obese *Lepr*^L536Hfs*^^6^ founder mouse were crossed with 129S6Ev. The underlying mutation was identified using QTL analysis and followed by whole genome sequencing (28). For the QTL analysis, 57 first backcross (BC) hybrids ([(*Lepr*^L536Hfs*6^ × 129S6Ev) F1 × *Lepr*^L536Hfs*^^6^] BC1) were genotyped genome wide with an 822 single nucleotide (SNP) panel by Taconic (Taconic Bioscience, NY). With these SNP genetic markers, QTL mapping of the phenotype body fat percentage was performed with the package R/qtl using Haley Knott regression (28). This identified an association of body fat percentage with a region on chromosome 4, markers flanking the 95%-confidence interval corresponding to region 4:70 Mb–110 Mb with a maximum LOD score of 5 at marker rs6271003. First backcross hybrids (BC1) identified monogenic, recessive Mendelian inheritance of the *Lepr*^L536Hfs*^^6^ phenotype (27).

### Whole genome sequencing

Two wt mice, two mice with the *Lepr*^L536Hfs*^^6^ phenotype, and one presumably heterozygous mouse (judged by offspring with *Lepr*^L536Hfs*^^6^ phenotype) were sequenced to identify the causative mutation of the *Lepr*^L536Hfs*^^6^ phenotype. Whole genome sequencing of the five mice was performed using paired-end sequencing (250 or 200 bp) on the Illumina HiSeq platform. Adaptor sequences were trimmed using cutadapt 1.9.1 (29) and the sequencing reads were aligned to the mouse reference genome mm38 with bwa 0.7.12 (30). Reads that mapped equally well to more than one genomic position were discarded. Reads were sorted using samtools 1.3.1 (31) and PCR duplicates removed with picard-tools-2.5.0 (http://broadinstitute.github.io/picard). Variants were called with the GATK pipeline version 3.6 (32) and annotated with the Variant Effect Predictor by Ensembl (33). Filtering the resulting VCF file in the QTL region at chromosome 4 to coding variants where *Lepr*^L536Hfs*^^6^ mice were homozygous, the presumably heterozygous mouse was heterozygous and wt mice carried the wt allele identified the *Lepr*^L536Hfs*^^6^ mutation (average sequencing depth of 88 reads across samples) as the only variant fulfilling those criteria.

### Genotyping

*Lepr*^L536Hfs*^^6^ genotyping was conducted with FastStart PCR Master (Roche, Mannheim, Germany), Primer (fw: CATGGCATTCACCCCACAGTA, rev: CTGGCTTTTCCCAAGATAC, Biomers, Ulm, Germany), and the program 95°C 15 min, 95°C 30 s, 60°C 30 s, 72°C 1 min (35 cycles), 72°C 10 min, 4°C forever. The PCR-products were sequenced with the same primers by Sanger sequencing and analyzed for the mutation with FinchTV (Geospiza, Inc., Version 1.4.0). Genotyping of *Lepr*^*db/db*^ mice was conducted according to Peng *et al.* (34).

### Phenotyping

In two setups, seven to eight mice of each mouse strain, *Lepr*^L536Hfs*^^6^ and *Lepr*^*db/db*^ of both sexes were studied from age of six weeks up to an age of 12 or 30 weeks of life. Female data are shown, male data are available in the supplemental material part. Body weight was recorded weekly, body composition (fat and lean mass) analysis was measured at week 6, 10, 20, and 28 using EchoMRI700™ instrument (Echo Medical Systems, Houston, TX). Intraperitoneal glucose tolerance tests (GTTs) and insulin tolerance tests (ITTs) were performed at the age of 12 and 24 weeks. GTT was performed after an overnight fast for 16 h by injecting 2 g/kg body weight glucose and measuring the blood glucose levels after tail vein incision at 0 (baseline), 10, 30, 60, and 120 min after injection. ITT was performed in random-fed animals by injecting 0.75 unit/kg body weight human regular insulin (40 units Actrapid; Novo Nordisk, Copenhagen, Denmark). Glucose levels were determined in blood collected from the tail tip immediately before and 15, 30, and 60 min after the intraperitoneal injection (35). Indirect calorimetry was conducted by a Calorimetry Module (PhenoMaster V5.9.9, TSE Systems, Bad Homburg, Germany) at an age of 11 weeks as described before (36). Daily food intake was performed within the Calorimetry Module or for one week at the age of 21 weeks and was calculated as the average intake of chow within the time stated. Serum lipid concentrations were measured after an overnight fast at an age of 12 and 30 weeks.

Mice were sacrificed at 12 or 30 weeks of age by an overdose of anesthetic (Isofluran, Baxter, Unterschleißheim, Germany) after fasting for 14–16 h. Body- (naso-anal-length) and tail length, body weight, blood sugar (FreeStyle Freedom light, Abbott Diabetes Care, Wiesbaden, Germany), rectal body temperature (TH-5, Thermalert Monitoring Thermometer, Clifton, NJ), and glycosylated hemoglobin (HbA1c) (COBAS 7000, Roche, Basel, Switzerland) were measured. Liver, subcutaneous (sc), and epigonadal (epi) adipose tissue (AT) were immediately removed, weighed, and related to the whole-body mass to obtain relative organ weights. Organs were immediately frozen by liquid nitrogen. Serum was collected at 12 and 30 weeks and frozen.

### Analytical Procedures

Adiponectin (Adiponectin mouse ELISA, Adipogen Life Sciences, Liestal, Switzerland), C-peptide (Mouse c-peptide ELISA, Alpco, Salem, NH), insulin (mouse insulin ELISA, Mercodia AB, Uppsala, Sweden), and leptin (Mouse/Rat Leptin, R&D systems Europe, Ltd., Abingdon, UK) were measured according to manufacturer’s protocol from mouse serum for both groups.

Liver samples were analyzed for glycogen content (Glycogen colorimetric Assay Kit II, Biovision, Inc., Milpitas, CA) and triglycerides (LabAssay™, Wako Pure Chemical Industries, Ltd., Osaka, Japan).

### Brain tissue preparation and immunohistochemical labeling

Mouse brain preparation was performed as previously described by Heiland *et al.* (37) at a postnatal age of 12 weeks. Two hours prior to brain preparation, mice were injected i.p. with 5 μg/g body weight of sterile leptin (Merck KGaA, Darmstadt, Germany) or vehicle (sterile 20 mM Tris/HCl, pH 8.0). The brains were cut at hypothalamus within the area of ventromedial hypothalamic nucleus (VMN) and paraventricular hypothalamic nucleus (PVN) as described previously (37). Primary antibody raised against pStat3 (cell signaling #9145, 1:500, overnight) (38) was used and visualized with secondary peroxidase-conjugated goat antibodies and Ni-DAB as substrate for 3–5 min.

### Histology, adipocyte size, and its distribution

ScAT, epiAT, and liver biopsies were fixed in 4% buffered formalin and imbedded in paraffin. Five micrometer sections were sliced with automatic microtome (HM 335S ThermoScientific, Rockford, IL) and stained with hematoxylin and eosin, pictures were taken and analyzed with Keyence BZ-X800 and Keyence BZ-X800 Analyzer 1.1.1.8 (Neu-Isenburg, Germany).

### Western blot

Proteins were isolated from tissue (after leptin i.p. injection (5 μg/g body weight) or 20 mM Tris/HCl injection for 2 h) with RIPA buffer and 3x Halt Protease and Phosphatase Inhibitor (Thermo Scientific, Rockford, IL) for hypothalamus in Precellys homogenizer (VWR, Darmstadt, Germany). After heating up in Lämmli buffer (Bio-Rad Laboratories Inc., Hercules, CA), samples were separated in 4%–15% Mini-Protean TGX Precast Gels (Bio-Rad Laboratories Inc., Hercules, CA), blotted on PVDF Hybond®-P Transfer Membrane (GE-Healthcare, Little Chalfont, GB) with semidry Fastblot B43 (Analytik Jena AG, Jena, Germany). The following primary antibodies were used: Stat3 (1:2,000, 5% nonfat dry milk, Cell Signaling Technology, #4904/#9132, 1:500, 5% BSA), Socs3 (1:500, 5% BSA, Cell Signaling Technology, #2923), Jak2 (1:1,000, 5% nonfat dry milk, cell signaling #3230), pAkt (1:1,000, 5% BSA, Cell Signaling Technology, #4051/#4058, 1:500), Akt (1:1,000, 5% BSA, Cell Signaling Technology, #4685/#9272, 1:500), p-Erk1/2 (1:500, 5% nonfat dry milk, Cell Signaling Technology, #9101/#4377), pErk1/2 (1:2,000, 5% nonfat dry milk, Cell Signaling Technology, #9102), Lepr (1:500, 5% BSA, R&D systems, AF497) and β-Actin (1:2,000, 5% BSA, Cell Signaling Technology, #3700/Sigma-Aldrich, A5441, 1:2000, 5% BSA) as loading control. Appropriate secondary antibody goat anti-rabbit IgG, HRP-conjugated (Cell Signaling Technology, CS7074, 1:2,000) or anti-mouse (Cell Signaling Technology, 7076P2, 1:2000) were used. Visualization was done using Pierce ECL Western Blotting Substrate (Thermo Fisher, Waltham, MA) and g:box system (Syngene, Cambridge, UK). For quantification, GeneTools software (Syngene, Cambridge, UK) was used.

### RNA isolation and gene expression

RNA was isolated from scAT, epiAT, and hypothalamus with RNeasy® Lipid Tissue Mini Kit and QIAzol in QIAcube (Qiagen, Hilden, Germany) according to manufacturer’s protocol. RNA was immediately reverse transcribed with Superscript II Reverse Transcriptase (Invitrogen/Thermo Fisher, Waltham, MA) and/or stored at –80°C. RNA from scAT and epiAT of *Lepr*^L536Hfs*^^6^, *Lepr*^*db/db*^, and wt were used for microarray analysis with a Clariom D chip conducted by the Core Unit DNA – Technologies (University of Leipzig, Germany). Gene expression in hypothalamus was measured with LightCycler® 480 SYBR Green I Master (Roche, Basel, Switzerland) and primer (biomers.net, Ulm, Germany) *Lep-Ra* fw: ACACTGTTAATTTCACACCAGAG, rev: AGTCATTCAAACCATAGTTTAGG (17); *Lep-Rb* fw: AATTGTTCCTGGGCACAAGGACTGA, rev: TTACTGGAGATGCAGTTGCTGACAG; total *Lepr* fw: GGCACCATTTCCGCTTCAAT, rev: ACCATCCAGTCTCTTGCTCC at LightCycler® 480 (Roche, Basel, Switzerland). Sequences for *Lep-Rb* and total *Lepr* were designed with pubmed primer blast and Primer3.

### Statistics

Statistical analyses were performed with the Prism 6.0 software (GraphPad Software, San Diego, CA). The following statistical tests were performed: two-tailed unpaired Student’s *t* test, two-way ANOVA with Tukey correction or multiple *t* test with correction by Holm-Sidak method with alpha = 5.00%. The used statistical test is indicated in each figure legend. Data are shown as mean ± SD or SEM, *P* values < 0.05 were considered as statistically significant. To develop QTL mapping and whole genome sequencing pipelines, bash and R programming languages were used (28).

### Study approval

All animal experiments were approved by the local ethics review committees and were performed according to local government guidelines of the Saxony animal protection law (Landesdirektion Sachsen, Approval No: TVV66/15, T02/19, TVV27/14, TVV10/20, Leipzig, Germany)

## Results

### A novel spontaneous *Lepr* gene mutation is associated with an obese phenotype

**Fig. 1:**
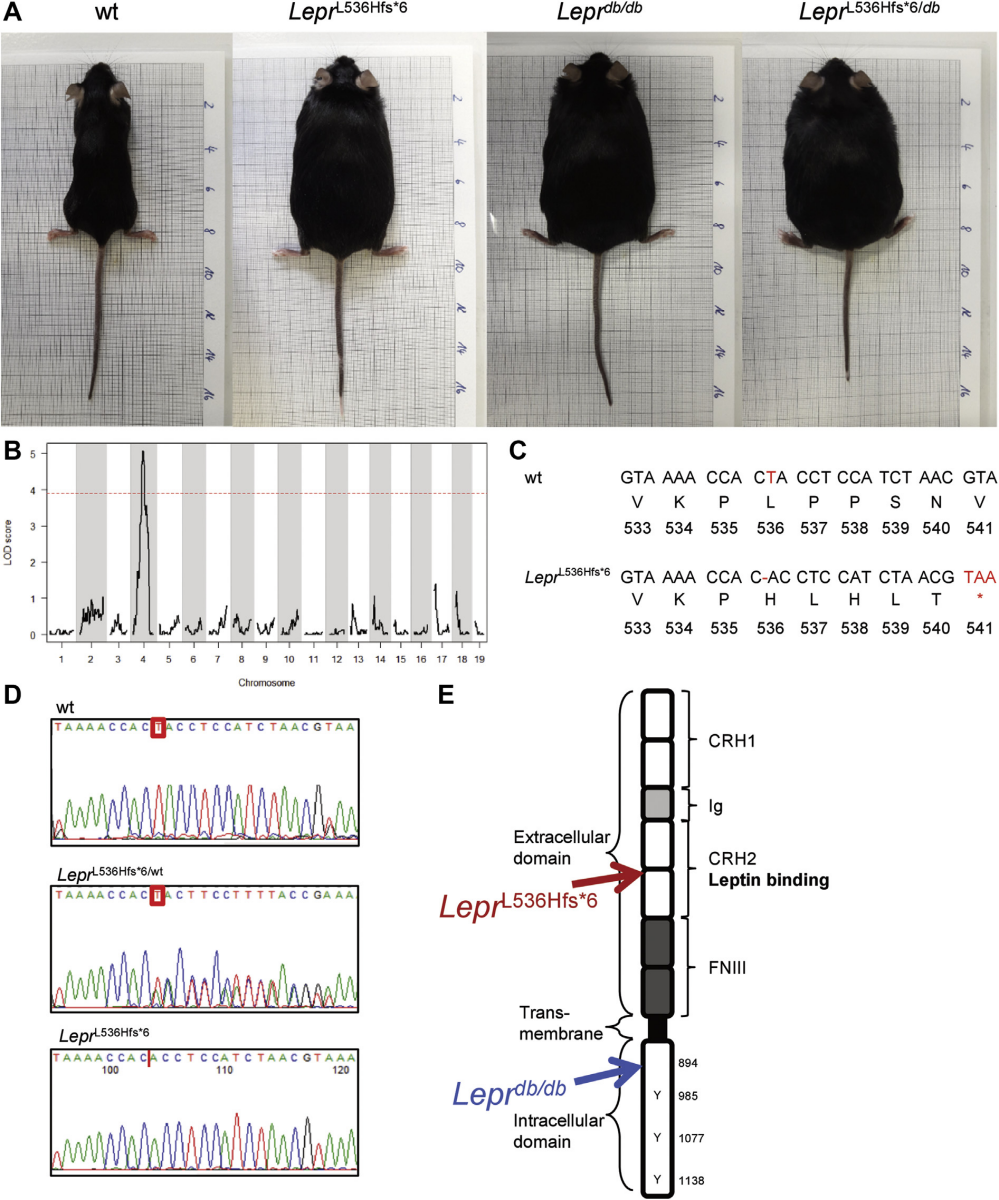
QTL analysis and *Lepr*^L536Hfs*^^6^ mutation. A: Pictures of 12-week-old female mice on mm scale. B: Quantitative trait locus (QTL) identification on chromosome 4 with R/qtl using Haley Knott regression (37). C: Nucleotide and amino acid sequences of the QTL region on chromosome 4 resulted in the identification of the point mutation at position 536 causing the novel *Lepr*^L536Hfs*^^6^ spontaneous *Lepr* mutant mice. The mutation results in a deletion in exon 11 which leads to a frame shift and stop codon within the next 5 aa. D: Exemplary sequence of a wt, a heterozygous *Lepr*^L536Hfs*^^6/wt^, and a homozygous *Lepr*^L536Hfs*^^6^ mouse. F: Schematic representation of the Lepr protein with highlighted positions for *Lepr*^L536Hfs*^^6^ and *Lepr*^*db/db*^ mutations as well as cytokine receptor homology (CRH1/2), Immunoglobulin domain (Ig), fibronectin III domain (FNIII).

We discovered a novel spontaneous single nucleotide deletion in the *Lepr*, which leads to a frame shift mutation and an early stop codon that causes the phenotype of our *Lepr*^L536Hfs*^^6^ mice. These mice exhibit differences in body weight and proportion (Fig. 1A, age- and gender-matched to wt, *Lepr*^*db/db*^, and compound heterozygous *Lepr*^L536Hfs*^^6/*db*^ mice).

In a backcross with C57BL/6NCrl mice, our colleagues at the Max Planck Institute of Experimental Medicine, Göttingen, noticed a spontaneous development of obesity in one offspring. Three animals, one obese female and two lean brothers, were transferred to our facilities. The obese female mouse was used to screen for known obese gene mutations. Since the obese female did not produce pups, according to the infertility of homozygous *Lepr*^L536Hfs*^^6^ mice, the lean brothers were crossed with C57BL/6N female mice. One of the two lean brothers was the founder and produced two litters, which were inbred with each other. Half of the inbred offspring were crossed with 129S6/EvNTac to generate F1 offspring. F1 were then backcrossed (BC) to males, which generated obese and lean pups [(*Lepr*^L536Hfs*^^6^ × 129S6Ev)F1 × *Lepr*^L536Hfs*^^6^] BC1. Genomic DNA samples from all 57 BC1 animals were analyzed using an 822 single nucleotide (SNP) panel. We mapped the body weight phenotype to a QTL locus on chromosome 4, 69 Mb–111 Mb (mm38, flanked by SNPs rs27880559 and rs28253902, Fig. 1B). The other half of the inbred strain was used to generate a new mouse line carrying the novel *Lepr* mutation. Heterozygous *Lepr* mutation carriers were backcrossed onto C57BL/6NTac background. The generated stable mouse line was then characterized and compared with the classical *Lepr*^*db/db*^ mice after backcrossing onto the same BL/6NTac background.

By sequencing the QTL region, a deletion of a thymidine (position 1607 bp beginning at start codon) in exon 11 was identified, which leads to a frame shift mutation followed by a stop codon in exon 11, predicting a protein of 540 aa instead of 1162 in wt *Lepr* (Fig. 1C and supplemental Fig. S1). Genotyping the offspring on C57BL/6N background revealed three different sequences accrued by Sanger sequencing as either wt, with thymidine, heterozygous *Lepr*^L536Hfs*^^6/wt^, with double sequences starting at point of deletion, or homozygous *Lepr*^L536Hfs*^^6^, with deleted thymidine, displayed in Fig. 1D. Looking at the whole Lepr, the location of the new mutation and the resulting stop codon is within the CRH2 leptin-binding site in contrast to the mutation in the *Lepr*^*db/db*^ mouse, which is located 350 aa apart (Fig. 1E).

### *Lepr*^L536Hfs*^^6^ mice are heavier and more obese than *Lepr*^*db/db*^ mice

**Fig. 2:**
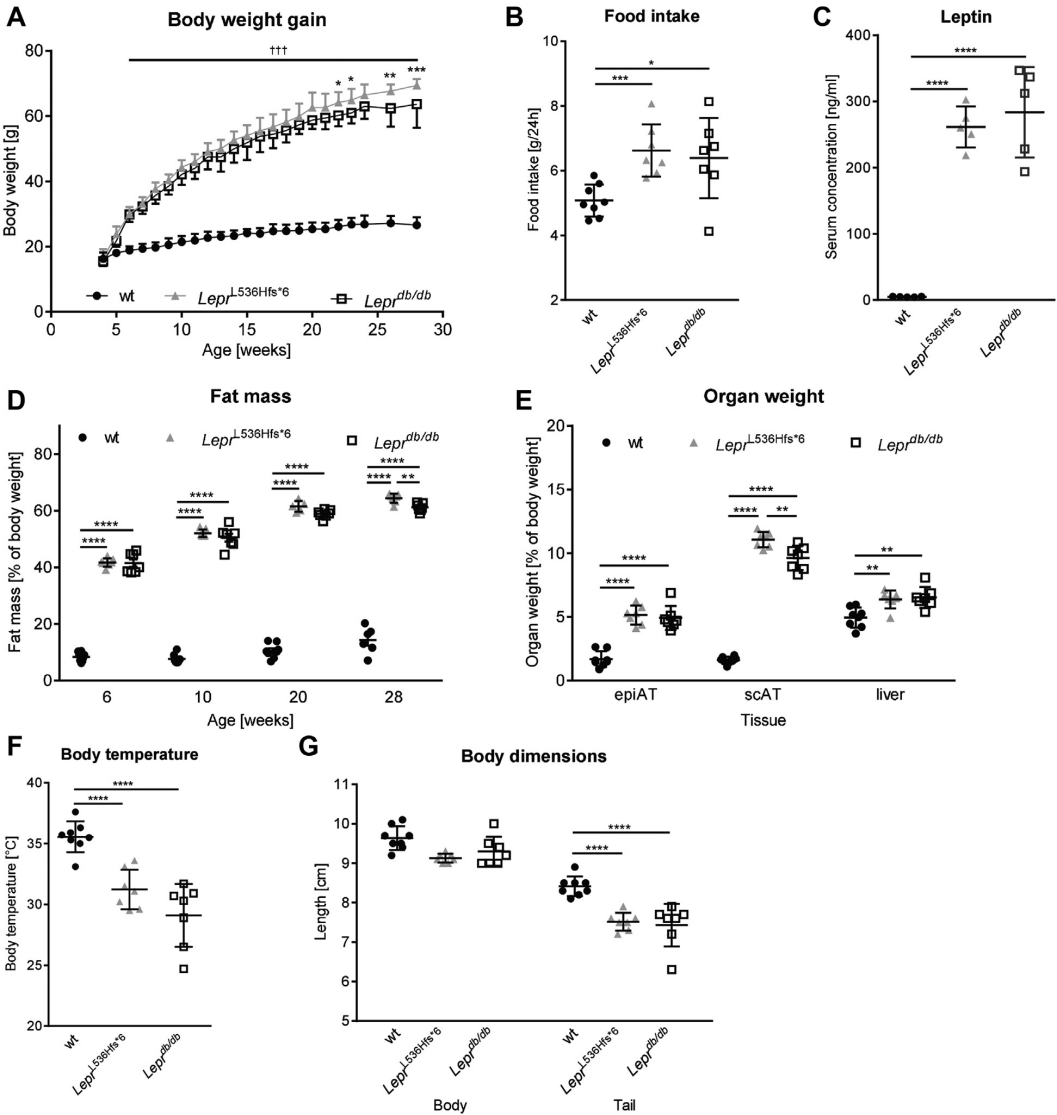
Spontaneous *Lepr*^L536Hfs*^^6^ mutation leads to obesity and increased food intake. Comparison of *Lepr*^L536Hfs*^^6^ to C57BL/6N wildtype (wt) littermates and *Lepr*^*db/db*^ mice with regard to (A) development of body weight from 4 to 28 weeks of age, significant values are marked with *Lepr^L536Hfs*^^6^ versus *Lepr*^*db/db*^, ^†^*Lepr*^L536Hfs*^^6^ and *Lepr*^*db/db*^ versus wt (n = 7–8, two-way ANOVA). B: Mean of food intake in 24 h at 21 weeks of age (n = 7–8, unpaired *t* test). C: Serum leptin concentrations measured by ELISA at 30 weeks of age (n = 5, unpaired *t* test). D: Fat mass determined by EchoMRI700™ instrument at the age of 6, 10, 20, or 28 weeks (n = 5–8, two-way ANOVA). E: Relative organ weights for epigonadal adipose tissue (epiAT), subcutaneous adipose tissue (scAT) and liver at 30 weeks of age (n = 7–8, two-way ANOVA). F: Body temperature (n = 7–8, unpaired *t* test), (G) naso-anal length and length of the tail in cm at 30 weeks of age (n = 7–8, two-way ANOVA). Data shown as mean ± SD, *P < 0.05, **P < 0.01, ***P < 0.001, ***P < 0.0001.

*Lepr*^L536Hfs*^^6^ mice and *Lepr*^*db/db*^ exhibited similar abnormal growth starting at an age of 6 weeks until the end of the observation period at 30 weeks of age compared with wt littermate controls (Fig. 2A). Differences between *Lepr*^L536Hfs*^^6^ and *Lepr*^*db/db*^ mice were observed in week 22 and 23 and from 26 weeks of age. Consistent with the higher body weight, food intake and serum leptin levels of *Lepr*^L536Hfs*^^6^ and *Lepr*^*db/db*^ mice were higher compared with wt mice (Fig. 2B, C). In addition, differences in fat mass were observed between wt and both obese strains starting in week 6 (wt 8.7%, *Lepr*^L536Hfs*^^6^ 41.7%, *Lepr*^*db/db*^ 41.3% fat of body weight, Fig. 2D). This trend continues until week 28 of age with a difference between *Lepr*^L536Hfs*^^6^ (64.4%) and *Lepr*^*db/db*^ (61.2%). Focusing on weight distribution (Fig. 2E), we found that subcutaneous AT (scAT) depot in *Lepr*^L536Hfs*^^6^ (11.1%) is significantly larger compared with *Lepr*^*db/db*^ (9.6%) and wt mice (1.6%). Relative liver and epigonadal AT (epiAT) weight was significantly increased in both obese strains compared with wt controls. Body core temperature (Fig. 2F) was not different between *Lepr*^L536Hfs*^^6^ and *Lepr*^*db/db*^ mice but decreased in comparison to lean wt littermate controls. Interestingly, both obesity mouse models had a shorter tail than their wt littermates (Fig. 1G).

### *Lepr*^L536Hfs*^^6^ mice develop impaired glucose metabolism

**Fig. 3:**
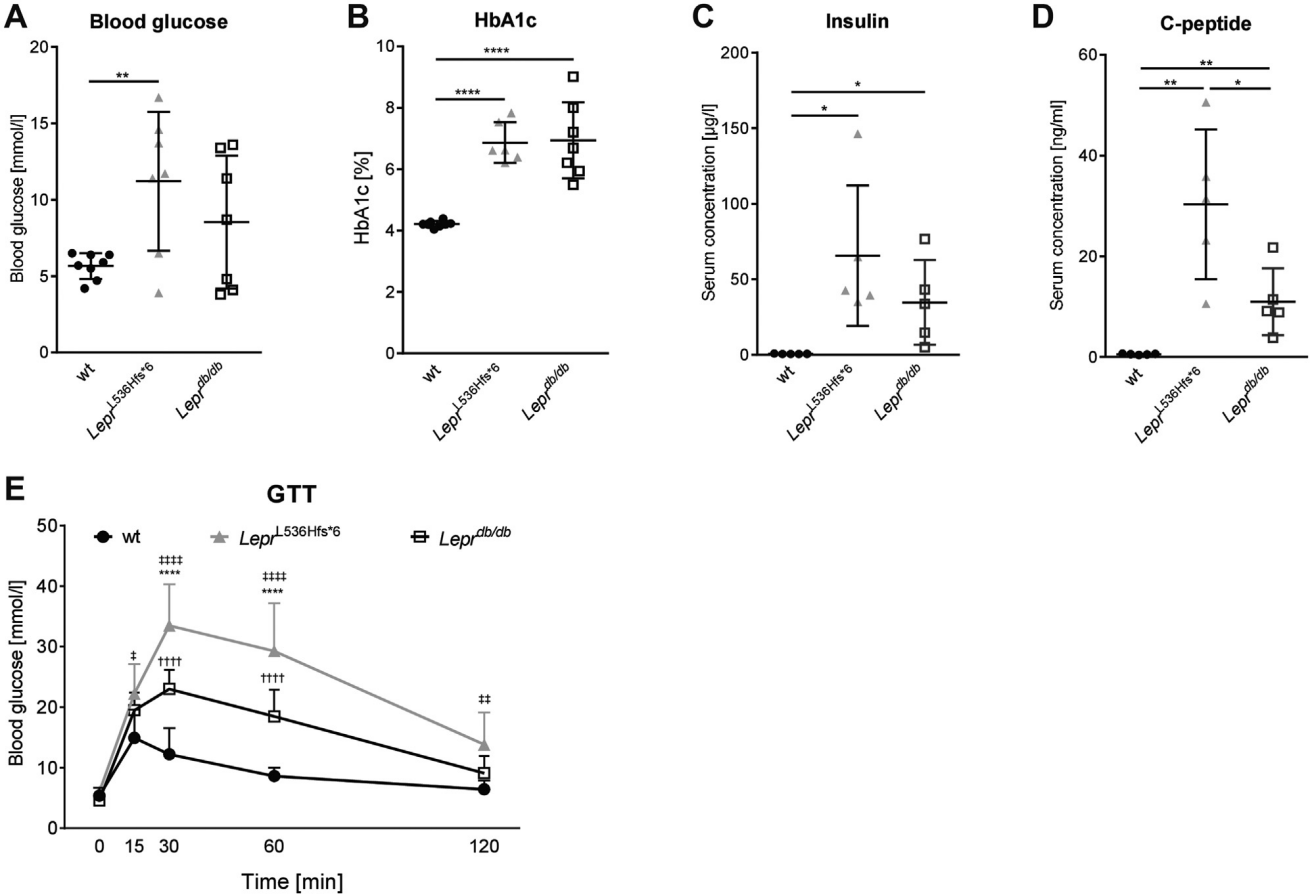
Comparison of glucose metabolism parameters between *Lepr*^L536Hfs*^^6^, wildtype, (wt) and *Lepr*^*db/db*^ mice. A: Blood glucose (n = 7–8, unpaired *t* test), (B) HbA1c (n = 6–8, unpaired *t* test) and results of ELISA for (C) insulin (n = 5, unpaired *t* test) and (D) c-peptide (n = 5, unpaired *t* test) at 30 weeks of age. E: Intra peritoneal glucose tolerance test (GTT) at 24 weeks of age (*Lepr^L536Hfs*^^6^ vs. *Lepr*^*db/db*^, ^‡^ *Lepr*^L536Hfs*^^6^ vs. wt, ^†^*Lepr*^*db/db*^ vs. wt, n = 7–8, two-way ANOVA), Data shown as mean ± SD, *P < 0.05, **P < 0.01, ***P < 0.001, ****P < 0.0001.

To determine the physiological consequences of *Lepr*^L536Hfs*^^6^ on glucose metabolism, we performed i.p. glucose tolerance test (GTT) at the age of 24 weeks, and monitored glycosylated hemoglobin (HbA1c), insulin and C-peptide levels at the end of the observation period. Fasting blood glucose levels as well as HbA1c were increased in *Lepr*^L536Hfs*^^6^ compared with wt mice (Fig. 3A, B). Fasting serum insulin concentrations were significantly higher in both obese mouse models compared with wt (Fig. 3C). Moreover, *Lepr*^L536Hfs*^^6^ mice had significantly elevated C-peptide levels compared with *Lepr*^*db/db*^ and wt mice (Fig. 3D). GTTs revealed an impaired glucose tolerance in both obese models compared with wt mice. Higher glucose levels were found in *Lepr*^L536Hfs*^^6^ compared with *Lepr*^*db/db*^ mice at 30 and 60 min after glucose injection (Fig. 3E). Only wt animals reached the initial blood glucose level while *Lepr*^L536Hfs*^^6^ mice stayed at 13.8 mmol/l and *Lepr*^*db/db*^ at 9.1 mmol/l after 120 min.

### Consequences of *Lepr*^L536Hfs*^^6^ mutation on lipid parameters and liver fat metabolism

As shown in table 1, circulating serum levels of triglycerides, total cholesterol, high-density lipoprotein (HDL), and low-density lipoprotein (LDL) cholesterol were significantly elevated in *Lepr*^L536Hfs*^^6^ compared with wt mice. Total cholesterol as well as HDL-cholesterol was significantly higher in *Lepr*^L536Hfs*^^6^ as in *Lepr*^*db/db*^ mice. Adiponectin serum concentrations of *Lepr*^L536Hfs*^^6^ and *Lepr*^*db/db*^ were not significantly different.

**Table 1:** Serum lipid concentrations.

Serum parameters	wt	*Lepr*^L536Hfs*^^6^	*Lepr*^*db/db*^
Triglycerides (mmol/l)	1.018 ± 0.335	**2.064 ± 0.438**^b^	1.392 ± 0.138
Total cholesterol (mmol/l)	2.288 ± 0.458	**7.526 ± 0.847**^a^^,^^b^	**5.798 ± 0.836**^c^
HDL-cholesterol (mmol/l)	1.790 ± 0.351	**5.644 ± 0.708**^a^^,^^b^	**4.452 ± 0.651**^c^
LDL-cholesterol (mmol/l)	0.384 ± 0.111	**1.326 ± 0.164**^b^	0.814 ± 0.228
Free fatty acids (mmol/l)	1.142 ± 0.336	1.854 ± 0.147	1.358 ± 0.384
Adiponectin (μg/ml)	149.592 ± 61.928	**26.704 ± 16.206**^b^	**31.457 ± 4.730**^c^

Data shown as mean ± SD, at an age of 30 weeks, n = 5, *P* < 0.05 are shown in bold, tested by unpaired *t* test (Adiponectin), two-way ANOVA (all others).

a *Lepr*^L536Hfs*^^6^ versus *Lepr*^*db/db*^.

b *Lepr*^L536Hfs*^^6^ versus wt.

c *Lepr*^*db/db*^ versus wt.

**Fig. 4:**
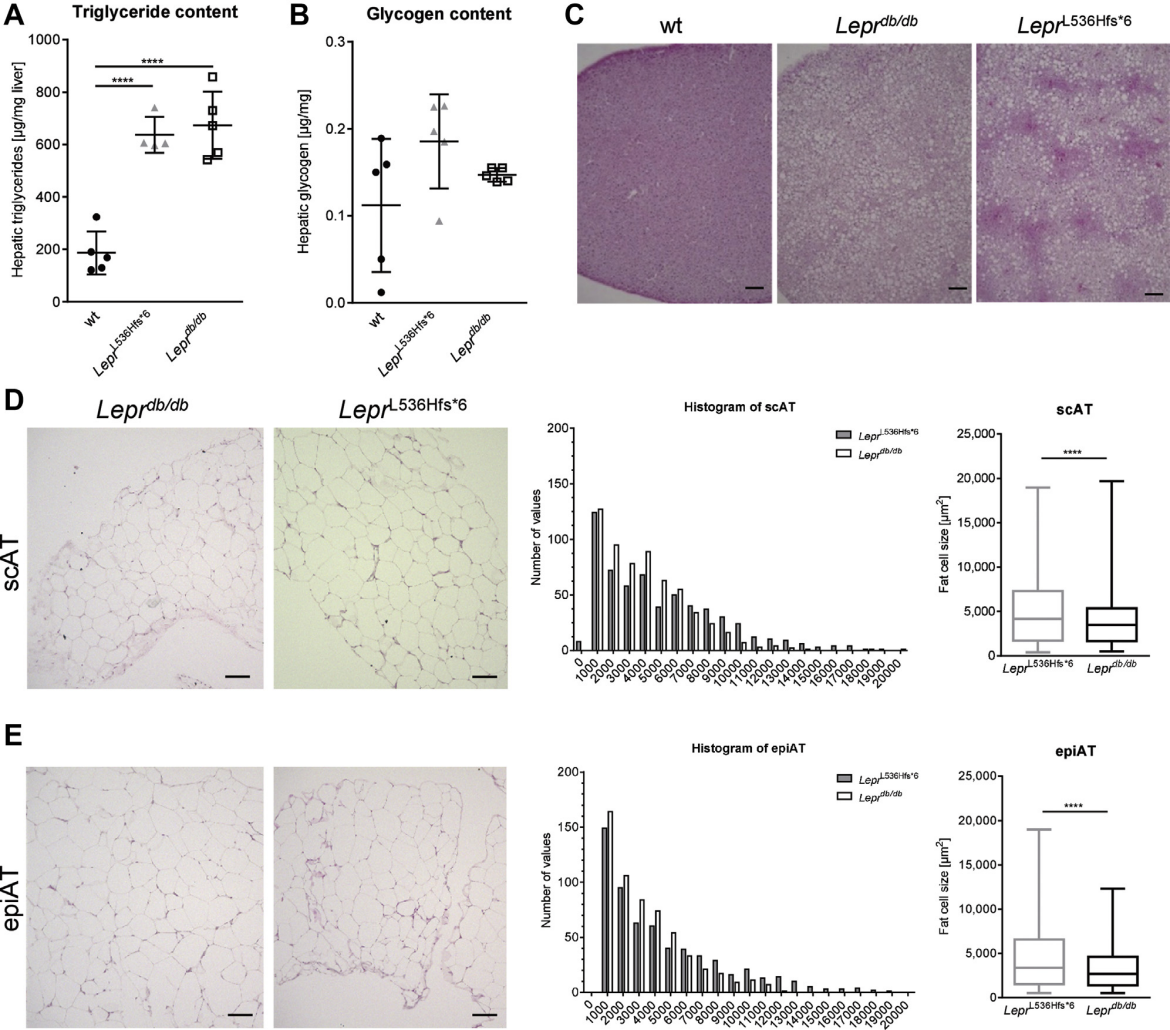
Consequences of the *Lepr*^L536Hfs*^^6^ mutation on liver and adipose tissue. Comparison of *Lepr*^L536Hfs*^^6^ to C57BL/6N wildtype (wt) and *Lepr*^*db/db*^ mice with regard to liver (A) triglyceride content (n = 4–5, unpaired *t* test, mean ± SD) and (B) glycogen content (n = 5, unpaired *t* test, mean ± SD). C: Histological differences as displayed in representative slides of liver stained with hematoxylin and eosin. Scale bars represent 100 μm. D–E: Comparison of adipose tissue between *Lepr*^L536Hfs*^^6^ and *Lepr*^*db/db*^ mice. D: Representative histological slides of subcutaneous adipose tissue (scAT) stained with hematoxylin and eosin. Scale bars represent 100 μm. Inguinal subcutaneous (sc) fat cell size distribution analyzed with Keyence BZ-X800 Analyzer and mean fat cell size calculated out of fat cell size (n = 600, unpaired *t* test, median ± min/max). E: Representative histological slides of epigonadal adipose tissue (epiAT) stained with hematoxylin and eosin. Scale bars represent 100 μm. EpiAT fat cell size distribution and mean fat cell size in epiAT from *Lepr*^L536Hfs*^^6^ compared with *Lepr*^*db/db*^ mice (n = 581–600, unpaired *t* test, median ± min/max).

Hepatic triglyceride content was significantly increased in both *Lepr*^L536Hfs*^^6^ (637 μg/mg tissue) and *Lepr*^*db/db*^ (674 μg/mg tissue) compared with wt mice (186 μg/mg tissue), while hepatic glycogen content was not different (Fig. 4A, B). Histological slides stained with hematoxylin and eosin (Fig. 4C) underline the higher hepatic liver fat accumulation seen in both, *Lepr*^*db/db*^ and *Lepr*^L536Hfs*^^6^ mice compared with wt. Histological analysis of scAT and epiAT illustrated an increased mean adipocyte size of *Lepr*^L536Hfs*^^6^ compared with *Lepr*^*db/db*^ in both fat depots (Fig. 4D, E).

### Energy expenditure and spontaneous activity are decreased in *Lepr*^L536Hfs*^^6^ mice

**Fig. 5:**
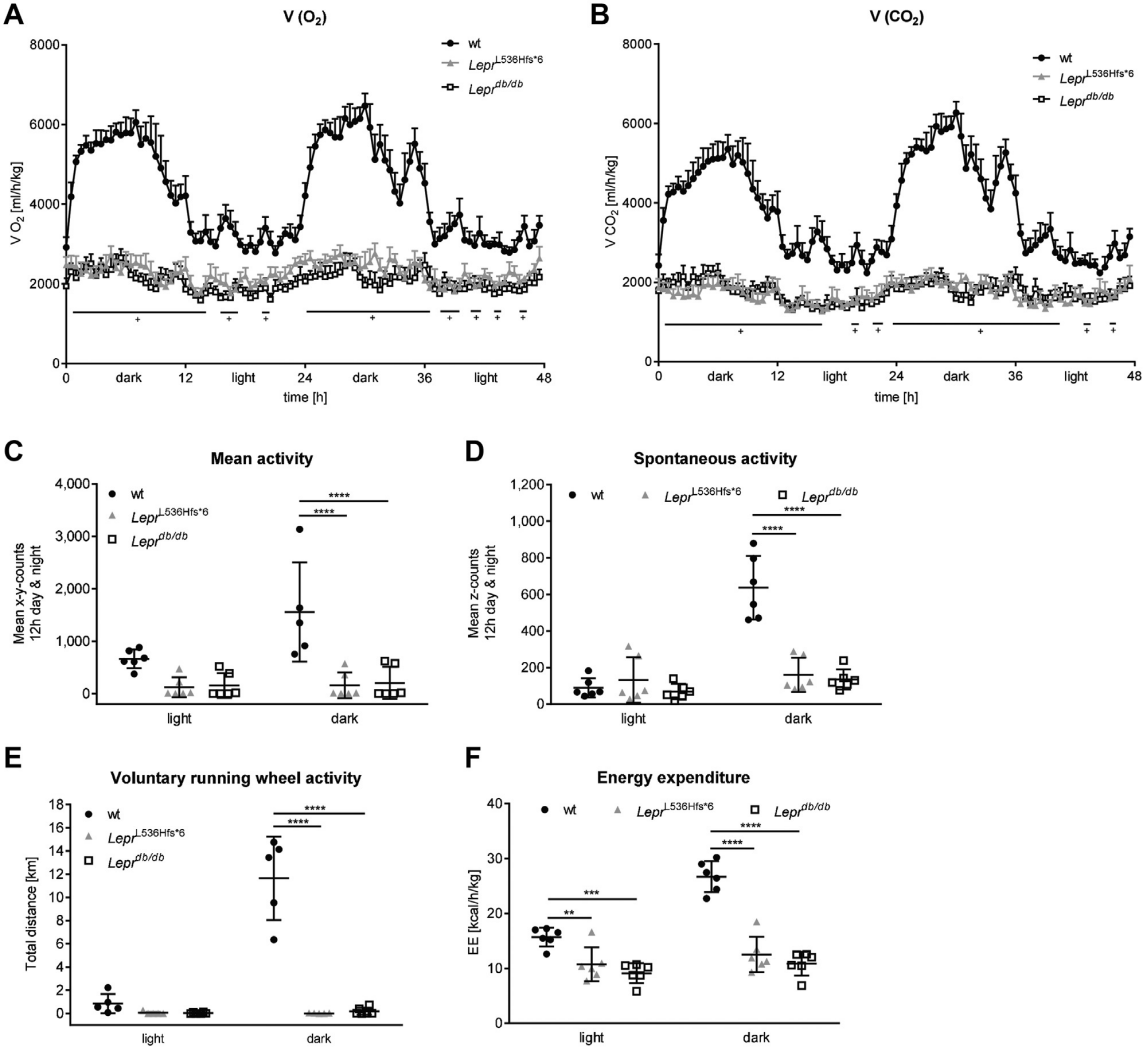
Characterization of energy metabolism in *Lepr*^L536Hfs*^^6^, wildtype, and *Lepr*^*db/db*^ mice. All mice were housed in a metabolic chamber for 72 h. Data of two consecutive dark and light phases (in total 48 h) after a 24 h adaptation phase. A: Oxygen consumption (V(O_2_), n = 5–6, two-way ANOVA) and (B) CO_2_ production (V(CO_2_), n = 5–6, two-way ANOVA) are significantly lower in both *Lepr*^L536Hfs*^^6^ and *Lepr*^*db/db*^ compared to wildtype (wt) mice independently of the light or dark phase (^†^*Lepr*^L536Hfs*^^6^ and *Lepr*^*db/db*^ vs. wt). C: Mean activity level (n = 5–6, two-way ANOVA), (D) Spontaneous activity (n = 6, two-way ANOVA), (E) voluntary running wheel activity (n = 5–6, two-way ANOVA), and (F) energy expenditure (EE, n = 6, two-way ANOVA) during the light and dark phase in *Lepr*^L536Hfs*^^6^, wt and *Lepr*^*db/db*^ mice. Data shown as mean ± SEM (A, B) or mean ± SD (C–F), *P < 0.05, **P < 0.01, ***P < 0.001, ****P < 0.0001.

Metabolic chamber analysis displayed differences between wt animals and both obese mouse models. Looking at the differences in activity and volume of oxygen consumption (V(O_2_)) as well as CO_2_ emission (V(CO_2_)) between light and dark phase, only wt animals had an active phase during the dark and a recovery phase during the light phase (Fig. 5A, B). In contrast, *Lepr*^L536Hfs*^^6^ and *Lepr*^*db/db*^ mice had a constantly low mean activity as well as a steady oxygen consumption and carbon dioxide emission in the light and in the dark phase indicating a disturbed circadian rhythm. Significantly higher volumes of O_2_ and CO_2_ were detected in wt mice compared with *Lepr*^L536Hfs*^^6^ and *Lepr*^*db/db*^*.*

Mean activity, measured by x-y-axis counts, spontaneous activity, measured by z-axis counts, and voluntary running wheel activity were decreased during the dark in *Lepr*^L536Hfs*^^6^ and *Lepr*^*db/db*^ mice compared with wt animals (Fig. 5C–E). In contrast to wt animals, both obese models represented lower energy expenditure during light and dark phase (Fig. 5F).

### Leptin signaling pathway

**Fig. 6:**
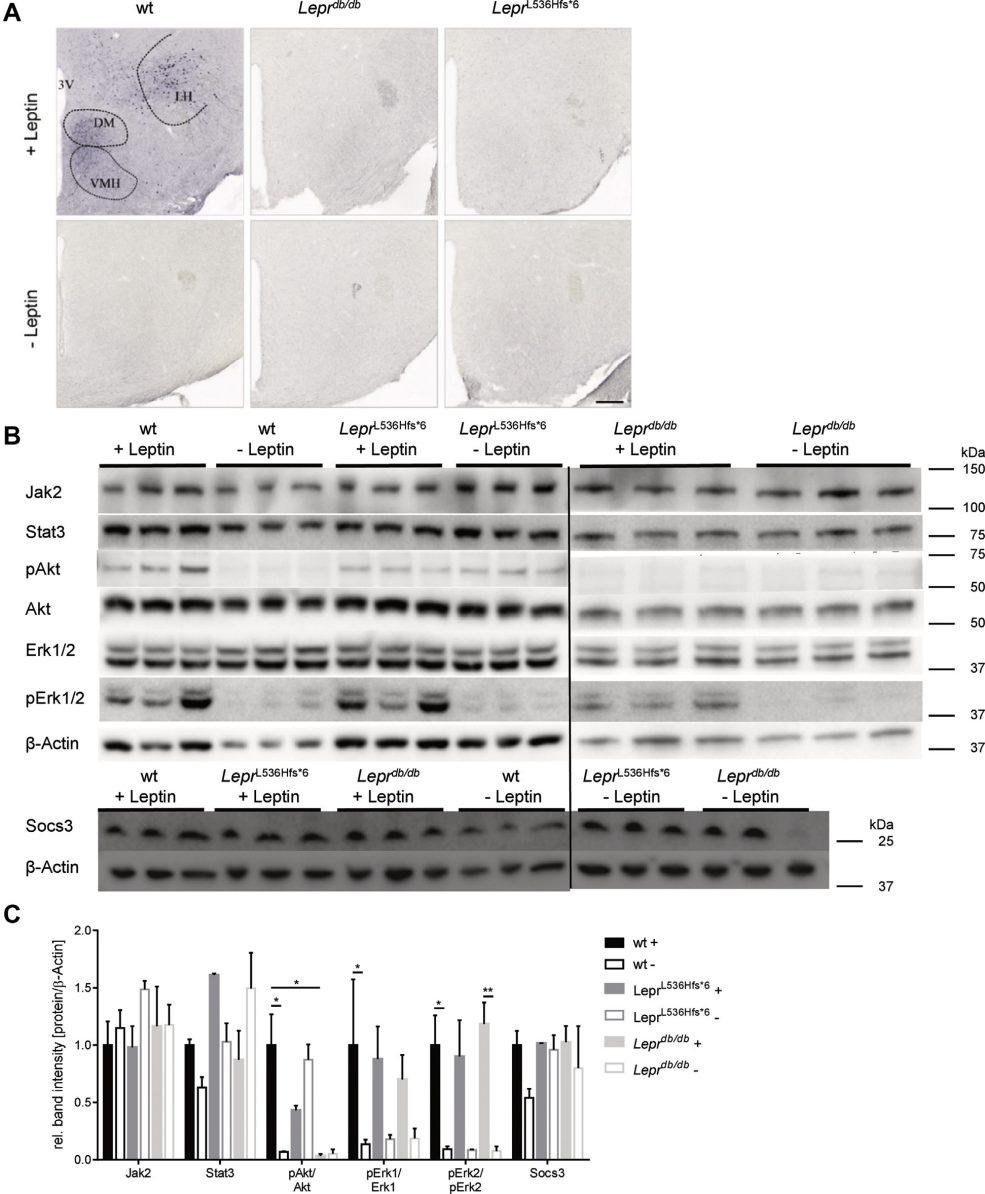
Characterization of hypothalamic signaling in *Lepr*^L536Hfs*^^6^, wildtype, and *Lepr*^*db/db*^ mice. A: Immunohistochemistry of pStat3 on brain slides. Wildtype (wt) +Leptin with pStat3 positive cells within VMH (ventromedial hypothalamus), DM (dorsomedial hypothalamic nucleus), and LH (lateral hypothalamic area), wt -Leptin, *Lepr*^L536Hfs*^^6^ +/−Leptin, and *Lepr*^*db/db*^ +/−Leptin without signal. Mice were fasted for 14–16 h prior to leptin injection (i.p. 5 μg/g body weight, +Leptin) or vehicle administration (−Leptin), 2 h later mice were sacrificed, brains were fixed by 4% PFA and removed, scale 200 μm. B: Induction of leptin signaling measured by western blot quantification of Jak2, Stat3, pAkt, Akt, Erk1/2, pErk1/2, and Socs3 in total hypothalamus protein. β-Actin served as loading control. Mice were fasted for 14–16 h prior to leptin injection (i.p. 5 μg/g body weight, +Leptin) or vehicle administration (−Leptin) and brains were removed 2 h later and hypothalamus was dissected out of the brain. Data shown as mean ± SEM, two-way ANOVA, n = 3/group, *P < 0.05.

The metabolic activity of leptin is mainly determined in the hypothalamus by binding to the Lepr and triggering Stat3 activation in neurons of the arcuate nucleus and ventromedial nucleus of the hypothalamus (39, 40). To determine leptin-Lepr signaling in the brain, *Lepr*^L536Hfs*^^6^, *Lepr*^*db/db*^, and wt control mice were fasted over night for ∼16 h prior to injection of leptin and sacrificed 2 h later for immunohistochemistry as previously published (38) or prepared for western blot. As shown in Fig. 6A, pStat3 positive cells were detected in the area of VMH (ventromedial hypothalamus), DM (dorsomedial hypothalamic nucleus), and LH (lateral hypothalamic area) of wt, whereas *Lepr*^*db/db*^ and *Lepr*^L536Hfs*^^6^ mice did not exhibit pStat3 positive nuclei, indicating a complete unresponsiveness to leptin in *Lepr*^L536Hfs*^^6^ mice.

Western blot data of Jak2, Stat3, and Socs3 showed no genotype-depended differences in protein concentration. After activation of the signal cascade *via* leptin stimulation, a 14.5-fold increase in the ratio of pAkt/Akt was measured in wt, while no change of the ratio was measured in *Lepr*^L536Hfs*^^6^ and *Lepr*^*db/db*^ mice. The ratios of pErk1/Erk1 and pErk2/Erk2 were also increased in wt and for *Lepr*^*db/db*^ mice of pErk2/Erk2 after leptin injection, while protein concentrations in *Lepr*^L536Hfs*^^6^ were not affected significantly (Fig. 6B, C).

### Compound heterozygosity of *Lepr*^L536Hfs*^^6/db^ results in higher body weight whole body fat mass and HbA1c levels

**Fig. 7:**
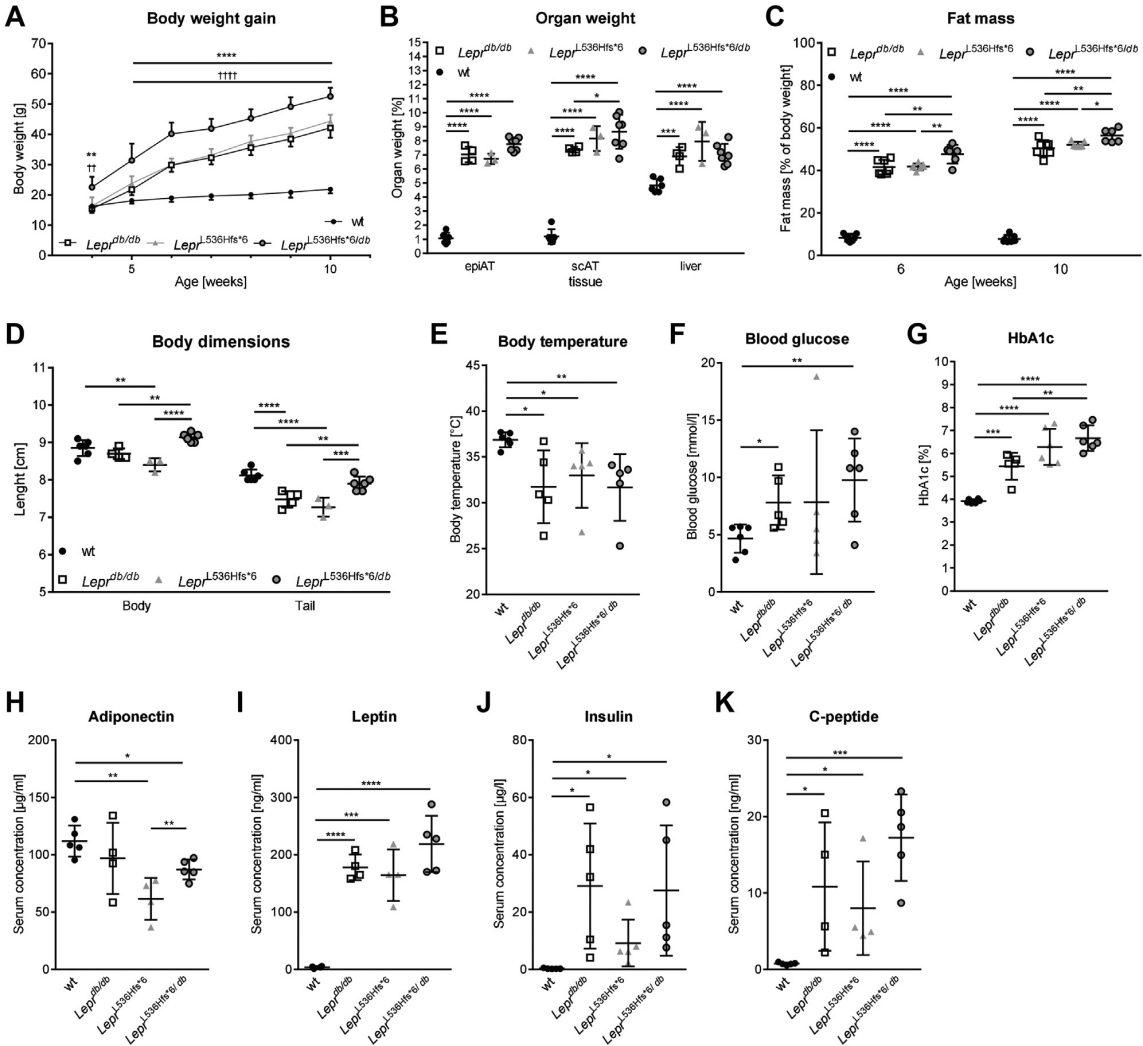
Phenotype of the compound heterozygous Lepr^L536Hfs*^^6/db^ mouse. Comparison of *Lepr*^L536Hfs*^^6/*db*^ to *Lepr*^L536Hfs*^^6^, wildtype (wt) and *Lepr*^*db/db*^ mice with regard to (A) body weight gain between 4 and 10 weeks of age (n = 6–7, *s*Lepr*^L536Hfs*^^6/*db*^ vs. *Lepr*^L536Hfs*^^6^ and *Lepr*^*db/db*^, ^†^*Lepr*^L536Hfs*^^6*/db*^ vs. wt, two-way ANOVA). B: Relative organ weights (n = 3–7, two-way ANOVA). C: Whole body fat mass at 6 and 10 weeks of age measured by EchoMRI700™ (n = 6–7, two-way ANOVA). D: Body length (naso-anal-length) as well as tail length (n = 3–6, two-way ANOVA). E: body temperature (n = 5–6, unpaired *t* test), (F) fasting blood glucose (n = 5–6, unpaired *t* test), (G) HbA1c (n = 5–6, unpaired *t* test). Serum levels of (H): adiponectin (n = 4–5, unpaired *t* test), (I) leptin (n = 4–5, unpaired *t* test), (J) insulin (n = 5, unpaired *t* test) and (K) C-peptide (n = 4–5, unpaired *t* test) at 12 weeks of age. Data shown as mean ± SD, *P < 0.05, **P < 0.01, ***P < 0.001.

The presence of two different mutant alleles at a particular gene locus, one on each chromosome of a pair is called compound heterozygous. Starting at 5 weeks until the end of the observation period, compound heterozygous *Lepr*^L536Hfs*^^6/*db*^ gained more weight compared with the three comparator genotypes (Fig. 7A). *Lepr*^L536Hfs*^^6/*db*^ had a higher scAT weight than *Lepr*^L536Hfs*^^6^, whereas relative epiAT and liver weights were indistinguishable between the obesity mouse models (Fig. 7B). Focusing on body composition, *Lepr*^L536Hfs*^^6/*db*^ animals had a higher whole-body fat mass at 6 and 10 weeks of age than both homozygous animals (Fig. 7C). Interestingly, both body weight and naso-to-anal length were higher in compound heterozygous mice than in *Lepr*^L536Hfs*^^6^ and *Lepr*^*db/db*^ mice (Fig. 7D). While body temperature (Fig. 7E) and fasting blood glucose (Fig. 7F) were not affected by the *Lepr* compound heterozygosity, HbA1c (Fig. 7G) was higher in *Lepr*^L536Hfs*^^6/*db*^ compared with *Lepr*^*db/db*^ mice at the age of 12 weeks. *Lepr*^L536Hfs*^^6/*db*^ exhibited lower cholesterol (supplemental Table S1) than *Lepr*^L536Hfs*^^6^ as well as higher adiponectin (Fig. 7H) and C-peptide (Fig. 7K) serum concentrations than *Lepr*^L536Hfs*^^6^. Leptin (Fig. 7I) and insulin serum concentrations (Fig. 7J) were not different across the obese genotypes.

### Lepr expression and Jak/Stat signaling in *Lepr*^L536Hfs*^^6/*db*^

**Fig. 8:**
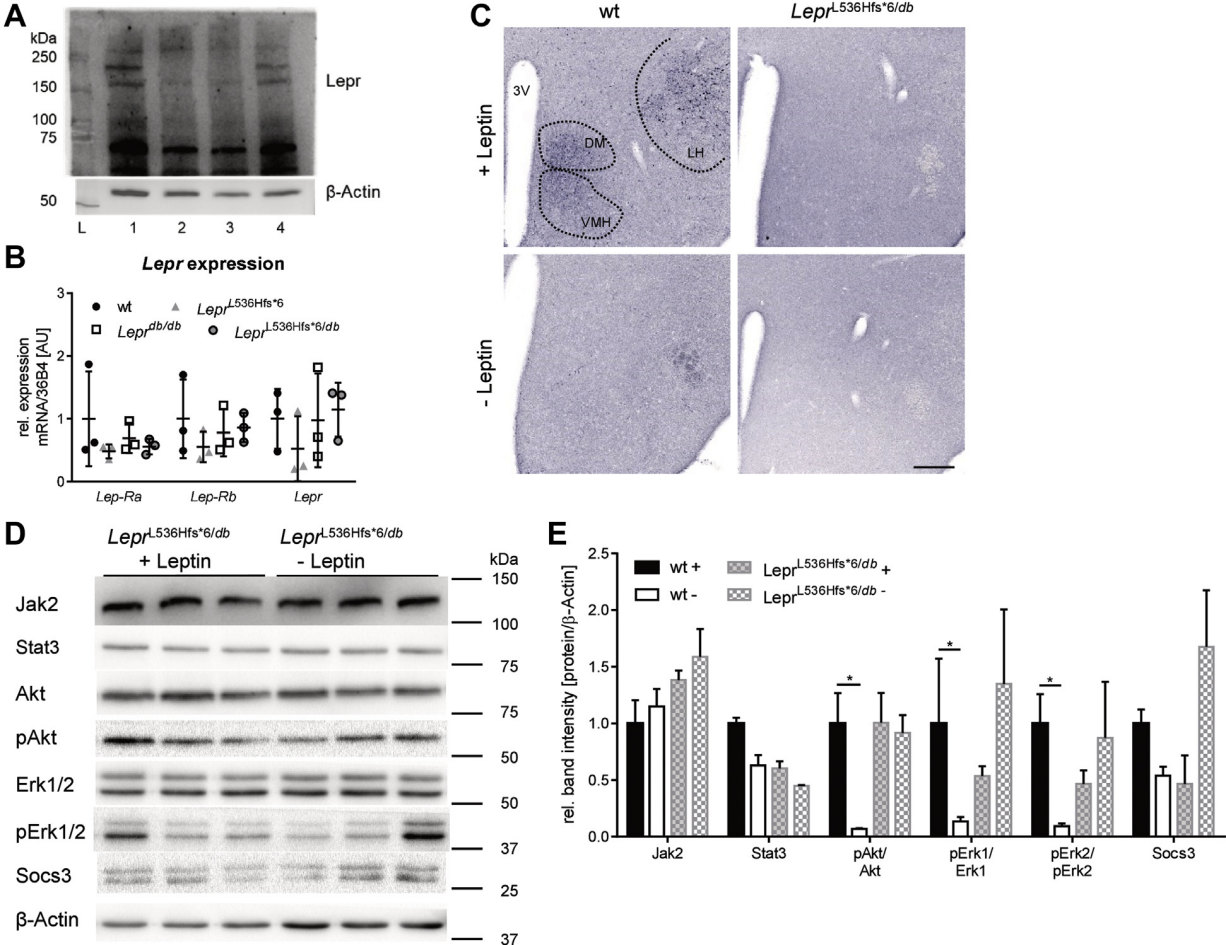
Characterization of hypothalamic Lepr expression and signaling in Lepr^L536Hfs*^^6/*db*^ and wildtype mice. A: Western blot of Lepr in hypothalamic protein with full-length receptor at ∼150 kDa, shedded protein at ∼70 kDa and β-Actin as loading control. L (Ladder), 1 (wildtype-wt), 2 (*Lepr*^L536Hfs*^^6^), 3 (*Lepr*^*db/db*^), 4 (*Lepr*^L536Hfs*^^6/*db*^). B: Relative mRNA expression of isoforms *Lep-Ra*, *Lep-Rb* and total *Lepr* within hypothalamus with *36B4* as housekeeping gene (n = 3, mean ± SD, two-way ANOVA). C: Immunohistochemistry of pStat3 on brain slides. Wildtype (wt) + Leptin with pStat3 positive cells within VMH (ventromedial hypothalamus), DM (dorsomedial hypothalamic nucleus), and LH (lateral hypothalamic area), wt −Leptin and *Lepr*^L536Hfs*^^6/*db*^ +/− Leptin without signal. Mice were fasted for 14–16 h prior to leptin injection (i.p. 5 μg/g body weight, +Leptin) or vehicle administration (−Leptin), 2 h later mice were sacrificed, brains were fixed by 4% PFA and removed, scale 200 μm. D: Induction of leptin signaling measured by western blot quantification of Jak2, Stat3, pAkt, Akt, Erk1/2, pErk1/2 and Socs3 in total hypothalamus protein. β-Actin served as loading control. Mice were fasted for 14–16 h prior to leptin injection (i.p. 5 μg/g body weight, +Leptin) or vehicle administration (−Leptin) and brains were removed 2 h later and hypothalamus was dissected out of the brain. Data shown as mean ± SEM, n = 3/group, two-way ANOVA.

Protein expression of full-length Lepr in hypothalamus was reduced in *Lepr*^L536Hfs*^^6^ and *Lepr*^*db/db*^ compared to wt levels (Fig. 8A), while gene expression of *Lep*-*Ra*, *Lep*-*Rb* and total *Lepr* was not different between the four different genotypes (Fig. 8B).

Immunohistochemical analysis of Stat3 phosphorylation in hypothalamus upon leptin stimulation displayed a lack of Stat3 activation like in the homozygous mouse models (Fig. 8C). Western blot data for proteins of the STAT signaling in the hypothalamus of *Lepr*^L536Hfs*^^6/*db*^ did not reveal a response to leptin treatment for all measured proteins of the Jak/Stat signaling cascade in comparison to *Lepr*^L536Hfs*^^6/*db*^ mice without leptin treatment (Fig. 8D, E).

### Characterization of energy metabolism in *Lepr*^L536Hfs*^^6^, *Lepr*^L536Hfs*^^6/*db*^, wt and *Lepr*^*db/db*^ mice

**Fig. 9:**
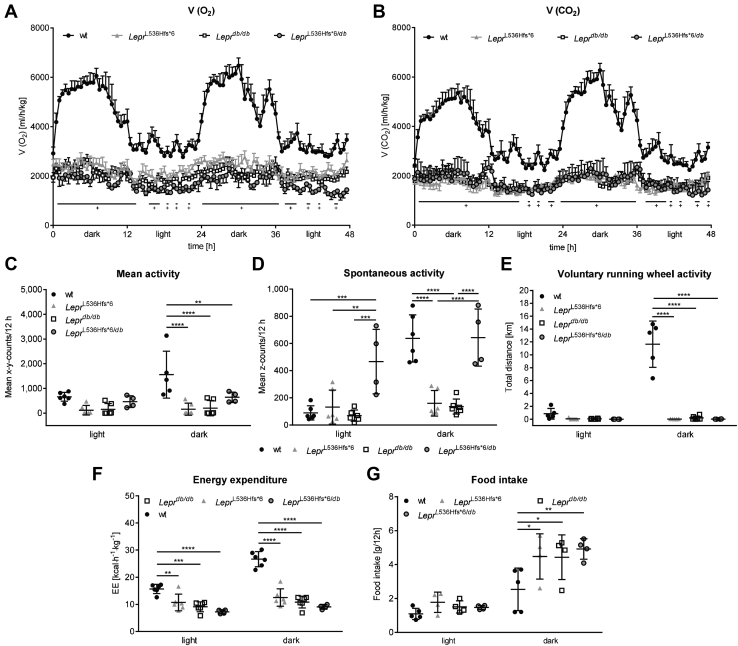
Characterization of energy metabolism in *Lepr*^L536Hfs*^^6^, *Lepr*^L536Hfs*^^6/*db*^, wildtype, and *Lepr*^*db/db*^ mice. All mice were housed in a metabolic chamber for 72 h. Data of two consecutive dark and light phases (in total 48 h) after a 24 h adaptation phase. A: Oxygen consumption (V(O_2_), n = 4–6, two-way ANOVA) and (B) CO_2_ production (V(CO_2_), n = 4–6, two-way ANOVA) are significantly lower in *Lepr*^L536Hfs*^^6^, *Lepr*^L536Hfs*^^6/*db*^, and *Lepr*^*db/db*^ compared with wildtype (wt) mice independently of the light or dark phase (^+^ *Lepr*^L536Hfs*^^6^, *Lepr*^L536Hfs*^^6/*db*^ and *Lepr*^*db/db*^ vs. wt). C: Mean activity (n = 4–6, two-way ANOVA), (D) spontaneous activity level (n = 4–6, two-way ANOVA), (E) voluntary running wheel activity (n = 4–6, two-way ANOVA), (F) energy expenditure (EE, n = 4–6, two-way ANOVA) and (G) Food intake (n = 4–6, two-way ANOVA) during the light and dark phase in *Lepr*^L536Hfs*^^6^, *Lepr*^L536Hfs*^^6/*db*^, wt and *Lepr*^*db/db*^ mice. Data shown as mean ± SEM (A, B) or mean ± SD (C–F), *P < 0.05, **P < 0.01, ***P < 0.001, ****P < 0.0001.

Analyzing the results of the metabolic chamber, *Lepr*^L536Hfs*^^6/*db*^ mice had comparable rate of oxygen consumption and carbon dioxide production as homozygous *Lepr*^L536Hfs*^^6^ and *Lepr*^*db*/*db*^ mice (Fig. 9A, B). Mean activity, voluntary running wheel activity and energy expenditure were also not significantly between *Lepr*^L536Hfs*^^6^, *Lepr*^L536Hfs*^^6/*db*^, and *Lepr*^*db/db*^ mice, while spontaneous activity of *Lepr*^L536Hfs*^^6/*db*^ mice was increased in light phase in comparison to all other analyzed genotypes, in dark phase it was only higher compared with *Lepr*^L536Hfs*^^6^ and *Lepr*^*db*/*db*^ mice (Fig. 9C–F). Food intake was elevated in the leptin receptor deficient mouse models compared with wt mice, but not significantly between *Lepr*^L536Hfs*^^6^, *Lepr*^L536Hfs*^^6/*db*^, and *Lepr*^*db/db*^ mice in dark phase. During light phase, there was no difference in food intake between the genotypes (Fig. 9G).

## Discussion

We discovered a novel spontaneous mutation within the *Lepr* gene, which causes severe obesity in homozygous mice. The 1 bp deletion on mouse chromosome 4 within exon 11 at position 1607 (counting from start codon) of the *Lepr* gene leads to a frameshift and a predicted early stop codon 5 aa later. Exon 11 is part of the cytokine receptor homology 2 (CRH2), which is the receptors’ main leptin-binding region (41). The early stop codon in this region leads to a decreased expression of full-length Lepr at the cell surface, leading to unfolded protein response (supplemental Fig. S2). In the hypothalamus, leptin-induced phosphorylation of Stat3 revealed that leptin signaling was differentially affected in neurons localized in the DM, VMN and LH of the mutant mice. Abrogated signaling in response to exogenous leptin was observed in the DM, VMN, and LH, suggesting that the *Lepr*^L536Hfs*^^6^ product predominates in these neurons and prevents responsiveness to leptin in this region. With the lower leptin binding and pStat3 signal transduction, energy balance is disturbed in *Lepr*^L536Hfs*^^6^ (4). We compared our new monogenic mouse model with the extensively characterized Lepr deficient *Lepr*^*db/db*^ mice. Since it is known that the background significantly influences metabolic parameters (42), we decided to backcross *Lepr*^*db/db*^ and *Lepr*^L536Hfs*^^6^ mice on the same BL/6NTac background to make sure that the observed phenotype of *Lepr*^L536Hfs*^^6^ mice is the result of differences in *Lepr* mutations and not background driven.

*Lepr*^L536Hfs*^^6^ mice are characterized by significantly higher body weight, hyperphagia, lower energy expenditure, and spontaneous activity compared with wt littermate controls. Our model underscores previous discoveries that genes that are critical for body weight regulation are mainly expressed in the hypothalamus and that mutated genes in monogenic forms of obesity play a key role in the leptin and melanocortin pathway (43, 44). In this context our monogenic mouse model of obesity considered confirmatory. However, using *Lepr*^L536Hfs*^^6*/db*^ mouse cross-breedings, we provide evidence that compound heterozygous mutation at the *Lepr* locus results in an even more pronounced obesity and impaired glucose metabolism phenotype than one would expect from the parental models. Our data may stimulate further human genetics research to test the hypothesis that compound heterozygous variants in the Leptin and or *LEPR* genes contribute to an increased obesity risk in subpopulations. Indeed, it has been recently shown that carriers of compound heterozygous mutations in the adenylate cyclase 3 (*ADCY3*) gene cause severe childhood obesity similar to *ADCY3* homozygous mutations (45).

In collaboration with Antje Körner, we recently found that heterozygosity of newly identified *LEPR* variants may contribute to the development of childhood morbid obesity (46). The finding that *Lepr*^L536Hfs*^^6^ mice have higher body weight and expandability of adipose tissue than *Lepr*^*db/db*^ mice further suggests that the genetic location of the mutation represents an important factor defining the phenotype. These differences between monogenic obesity models affecting the same gene at different loci are also reflected at the level of obesity-related metabolic alterations.

*Lepr*^L536Hfs*^^6^ mice have significantly higher fasting glucose, HbA1c, fasting insulin and C-peptide serum concentrations than wt mice. In comparison to other mouse models with mutations in extracellular parts of Lepr, *Lepr*^L536Hfs*^^6^ have worsened glucose tolerance than db^3J^ (with a peak at app. 21 mmol/l at age of 2 months) and a similar glucose dynamic as *Lepr*^G506S^ (at the age of 20 weeks) (11, 47). Although *Lepr*^G506S^, db^3J^, and our *Lepr*^L536Hfs*^^6^ have in common that Lepr mutations are located in the extracellular part (11, 47). Our model suggests that the exact variant may have an influence on glucose and insulin response, which seems to be distinct to each model. Interestingly, circulating C-peptide was 2-fold higher in *Lepr*^L536Hfs*^^6^ mice compared with *Lepr*^*db/db*^ mice suggesting that different *Lepr* mutations might affect beta cell function distinctly. However, the exact mechanisms underlying these differences are not clear. Based on the altered leptin signaling observed in *Lepr*^L536Hfs*^^6^ mice, we speculate that worse glucose tolerance results from diminished function of neurons in the VMN (48). Although, we cannot definitively rule out an indirect effect caused by the different body composition and higher scAT fat mass in *Lepr*^L536Hfs*^^6^ mice compared with *Lepr*^*db/db*^ mice, we hypothesize that elevated scAT fat mass may contribute to differences in glucose tolerance by differences in adipokine secretion patterns.

Interestingly, *Lepr*^L536Hfs*^^6^ mice did not have significantly altered leptin or adiponectin serum concentrations compared with *Lepr*^*db/db*^ mice. We can therefore only hypothesize that other adipokines or metabolites may contribute to the observed differences in glucose metabolism parameters between *Lepr*^L536Hfs*^^6^ and *Lepr*^*db/db*^ mice.

Importantly, spontaneous *Lepr* knock-down models may differ in the extent of how leptin mediated signaling, leptin binding, receptor expression on cell surfaces, receptor dimerization and activation are affected by a specific mutation. In a previous human study, it has been shown that even despite computational structure prediction suggested impaired leptin binding for all LEPR variants, biological experiments revealed important mutation-related differences in leptin binding or LEPR cell surface expression (46). Of the five Lepr isoforms, only the long Lepr isoform (Lep-Rb) has a functional intracellular domain, which mediates leptin signaling (9, 10). Conformational changes within the leptin-Lepr-complex initiate Jak2 autophosphorylation followed by phosphorylation of three tyrosine residues (Y985, Y1077, and Y1138) only present in the intracellular domain of Lep-Rb. Phosphorylation of Y1138 causes recruitment of signal transducer and activator of transcription (Stat3) to the Lep-Rb and Jak2 complex (26). Activation of Stat3 signaling pathway has been shown to mediate the effects of leptin on melanocortin production and energy homeostasis (25). In both *Lepr*^L536Hfs*^^6^ and *Lepr*^*db/db*^ mice, we could not detect pStat3 positive cells upon leptin treatment in VMH, DM, and LH, indicating a complete unresponsiveness to leptin upon this mutation. In addition, *Lepr*^L536Hfs*^^6^ have lower body temperature and energy expenditure, in accordance with other published *Lepr* mutated obese mouse models (49). Moreover, *Lepr*^L536Hfs*^^6^ may be considered as a model for fatty liver disease and impaired lipid metabolism due to the fact that the phenotype showed is similar to other reported fatty liver disease animal models (50).

In humans, the first family with a *LEPR* mutation was identified by Clement *et al.* (12). The prevalence of pathogenic *LEPR* mutations in a cohort with severe, early-onset obesity was 3% (51). In the homozygous mutation subjects, a truncation of the receptor before the transmembrane domain completely abolishes leptin signaling, leading to a form of massive obesity similar to that seen with leptin deficiency, along with significant growth retardation and central hypothyroidism (12). Patients with *LEPR* mutations typically develop very high circulating leptin levels. Chromatography of circulating leptin revealed that the hormone was bound to the truncated LEPR, leading to an increased plasma leptin half-life, although AT leptin expression correlates with fat mass (12). Therefore, the full knockout of the leptin pathway in humans was not responsible for the compensatory hypersecretion of leptin (52). Interestingly, the two subjects with the *LEPR* mutation in the study of Clement *et al.* neither developed type 2 diabetes nor dyslipidemia suggesting that mechanisms compensating for impaired leptin signaling are not entirely translatable between mice and men (12).

Compound heterozygous mutations are rarely found for *LEPR* and only seven different mutations in eight cases are described in humans up to now (46, 51, 53, 54, 55, 56). All of them had early onset of extreme obesity, seven had hyperphagia, three had altered growth and elevated leptin levels, two had hypogonadotropic hypogonadism, dyslipidemia, and hyperinsulinemia (46, 51, 53, 54, 55, 56). A study of Huvenne *et al.* analyzed 535 patients with severe obesity, only three of them had compound heterozygous mutations (54). These subjects probably had a truncated and/or damaged protein which results in a short LEPR only consisting of the extracellular part (54). We generated a genotype similar to this human example (36) by a crossbreeding strategy. We aimed to test the hypothesis that *Lepr*^L536Hfs*^^6/*db*^ mice will develop a phenotype distinct to that of the parental carriers of the homozygous mutations. *Lepr*^L536Hfs*^^6/*db*^ results in a deletion of the transmembrane and intracellular part of Lepr. Unexpectedly, *Lepr*^L536Hfs*^^6/*db*^ mice develop even more pronounced body weight gain upon hyperphagia, adiposity, and impairment of glucose metabolism than homozygous *Lepr*^L536Hfs*^^6^ and *Lepr*^*db/db*^ mice.

In contrast to humans with compound heterozygous *LEPR* mutations (46, 54), *Lepr*^L536Hfs*^^6/*db*^ mice do not have high circulating leptin levels at the age of 12 weeks. The higher extent of obesity and impairment of glucose metabolism in *Lepr*^L536Hfs*^^6/*db*^ compared with parental homozygous *Lepr* mutations cannot easily been explained, because both mutations are supposed to result in a functional knockout of the Lepr. In *Lepr*^*db*J/*db*J^ mice, there is no Lep-Rb protein produced and the transcript that should encode the *Lep-Rb* isoform is replaced by a novel transcript encoding the *Lep-Ra* isoform (9). Based on this assumption, *Lepr*^L536Hfs*^^6^ mice should either produce no Lepr protein at all or only a soluble truncated version, because *Lepr*^*db/db*^ used as controls in our study can still produce the *Lep-Ra* isoform. However, although we could confirm that *Lepr*^*db/db*^ indeed produce the *Lep-Ra* isoform, both *Lepr*^L536Hfs*^^6^ and *Lepr*^L536Hfs*^^6/*db*^ displayed *Lep-Ra* gene expressions that were indistinguishable to those produced by *Lepr*^*db/db*^ mice. It has been proposed that the long intracellular Lepr domain determines intracellular signal transduction and that functional absence of this Lepr form leads to the severe obese phenotype found in *Lepr*^*db*J/*db*J^ mice (9). Despite the fact that we did not find significant differences in *Lepr* isoform gene expression between the genotypes, we hypothesize that forced AAV-vector mediated expression of the short *Lep-Ra* isoform in *Lepr*^L536Hfs*^^6^ mice could recapitulate the exacerbated phenotype of *Lepr*^L536Hfs*^^6/*db*^ compound heterozygous mice.

We cannot exclude that both homozygous mutations result in an incomplete loss of Lepr despite the fact that soluble Lepr was not detectable by ELISA and western blot data suggest a lower concentration of long Lepr in homozygous obese mice compared with wt. Incomplete deletion of the Lepr or residual signaling activity of Lepr fragments may account for phenotype differences between *Lepr*^L536Hfs*^^6^ and *Lepr*^*db/db*^ mice that are reduced in the *Lepr*^L536Hfs*^^6/*db*^ model.

Taken together, we identified a novel spontaneous *Lepr* mutation, which causes obesity, hyperphagia, and impaired glucose metabolism similar to the well-described *Lepr*^*db/db*^ (13, 14) or other Lepr knockout mouse models (11).

## Conclusion

Beyond the known associations between *Lepr* mutations and a severely obesity phenotype, our study suggests that the phenotype of monogenic Lepr deficient mice depends on the molecular localization of the *Lepr* mutation. This assumption is further supported by the phenotype of compound heterozygous *Lepr*^L536Hfs*^^6/*db*^ mice that developed more severe obesity and hyperglycemia than the parental carriers of homozygous mutations. Although our model does not reveal a new obesity mechanism, *Lepr*^L536Hfs*^^6^ and *Lepr*^L536Hfs*^^6/*db*^ may serve the scientific community as an additional model to study mechanisms of extreme adipose tissue expandability and mechanisms how heterozygosity in candidate genes of obesity may worsen the phenotype. The latter model might be of particular interest to better understand how allelic and locus heterogeneity affects variation in human genotype-phenotype associations.

## Data availability

Data are available on request to Nora Klöting (Helmholtz Institute for Metabolic, Obesity and Vascular Research (HI-MAG), nora.kloeting@helmholtz-muenchen.de)

## Supplemental data

This article contains supplemental data.

## Conflict of interest

The authors declare that they have no conflicts of interest with the contents of this article.
