# Supplementary material for: A novel compound heterozygous leptin receptor mutation causes more severe obesity than in Leprdb/db mice
Source: J Lipid Res. 2021 Aug 11;62:100105. doi: 10.1016/j.jlr.2021.100105 (PMC8450258; doi:10.1016/j.jlr.2021.100105)
Supplement: Supplemental data [file mmc1.pptx]

## Slide 1
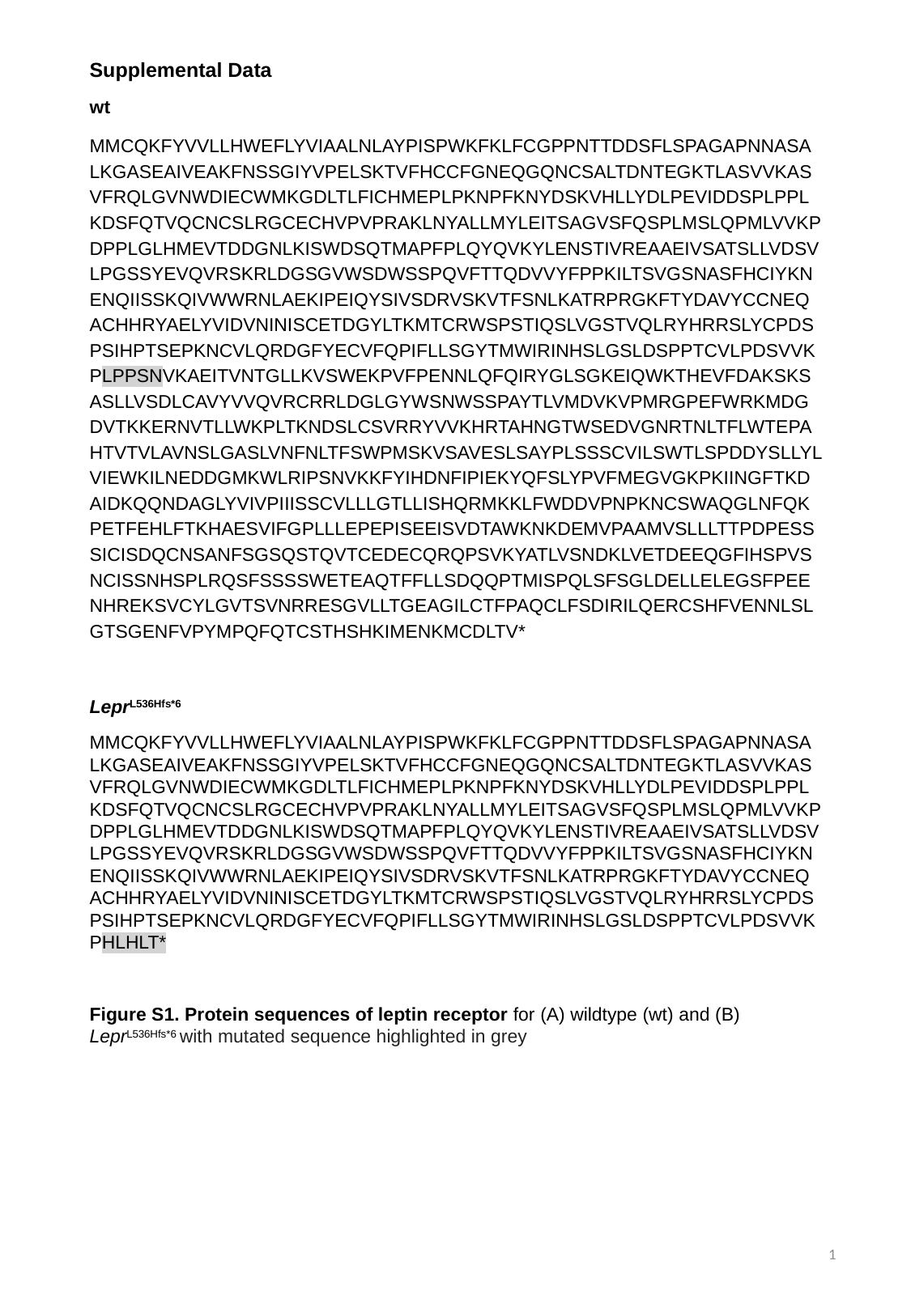

Supplemental Data
wt
MMCQKFYVVLLHWEFLYVIAALNLAYPISPWKFKLFCGPPNTTDDSFLSPAGAPNNASALKGASEAIVEAKFNSSGIYVPELSKTVFHCCFGNEQGQNCSALTDNTEGKTLASVVKASVFRQLGVNWDIECWMKGDLTLFICHMEPLPKNPFKNYDSKVHLLYDLPEVIDDSPLPPLKDSFQTVQCNCSLRGCECHVPVPRAKLNYALLMYLEITSAGVSFQSPLMSLQPMLVVKPDPPLGLHMEVTDDGNLKISWDSQTMAPFPLQYQVKYLENSTIVREAAEIVSATSLLVDSVLPGSSYEVQVRSKRLDGSGVWSDWSSPQVFTTQDVVYFPPKILTSVGSNASFHCIYKNENQIISSKQIVWWRNLAEKIPEIQYSIVSDRVSKVTFSNLKATRPRGKFTYDAVYCCNEQACHHRYAELYVIDVNINISCETDGYLTKMTCRWSPSTIQSLVGSTVQLRYHRRSLYCPDSPSIHPTSEPKNCVLQRDGFYECVFQPIFLLSGYTMWIRINHSLGSLDSPPTCVLPDSVVKPLPPSNVKAEITVNTGLLKVSWEKPVFPENNLQFQIRYGLSGKEIQWKTHEVFDAKSKSASLLVSDLCAVYVVQVRCRRLDGLGYWSNWSSPAYTLVMDVKVPMRGPEFWRKMDGDVTKKERNVTLLWKPLTKNDSLCSVRRYVVKHRTAHNGTWSEDVGNRTNLTFLWTEPAHTVTVLAVNSLGASLVNFNLTFSWPMSKVSAVESLSAYPLSSSCVILSWTLSPDDYSLLYLVIEWKILNEDDGMKWLRIPSNVKKFYIHDNFIPIEKYQFSLYPVFMEGVGKPKIINGFTKDAIDKQQNDAGLYVIVPIIISSCVLLLGTLLISHQRMKKLFWDDVPNPKNCSWAQGLNFQKPETFEHLFTKHAESVIFGPLLLEPEPISEEISVDTAWKNKDEMVPAAMVSLLLTTPDPESSSICISDQCNSANFSGSQSTQVTCEDECQRQPSVKYATLVSNDKLVETDEEQGFIHSPVSNCISSNHSPLRQSFSSSSWETEAQTFFLLSDQQPTMISPQLSFSGLDELLELEGSFPEENHREKSVCYLGVTSVNRRESGVLLTGEAGILCTFPAQCLFSDIRILQERCSHFVENNLSLGTSGENFVPYMPQFQTCSTHSHKIMENKMCDLTV*
LeprL536Hfs*6
MMCQKFYVVLLHWEFLYVIAALNLAYPISPWKFKLFCGPPNTTDDSFLSPAGAPNNASALKGASEAIVEAKFNSSGIYVPELSKTVFHCCFGNEQGQNCSALTDNTEGKTLASVVKASVFRQLGVNWDIECWMKGDLTLFICHMEPLPKNPFKNYDSKVHLLYDLPEVIDDSPLPPLKDSFQTVQCNCSLRGCECHVPVPRAKLNYALLMYLEITSAGVSFQSPLMSLQPMLVVKPDPPLGLHMEVTDDGNLKISWDSQTMAPFPLQYQVKYLENSTIVREAAEIVSATSLLVDSVLPGSSYEVQVRSKRLDGSGVWSDWSSPQVFTTQDVVYFPPKILTSVGSNASFHCIYKNENQIISSKQIVWWRNLAEKIPEIQYSIVSDRVSKVTFSNLKATRPRGKFTYDAVYCCNEQACHHRYAELYVIDVNINISCETDGYLTKMTCRWSPSTIQSLVGSTVQLRYHRRSLYCPDSPSIHPTSEPKNCVLQRDGFYECVFQPIFLLSGYTMWIRINHSLGSLDSPPTCVLPDSVVKPHLHLT*
Figure S1. Protein sequences of leptin receptor for (A) wildtype (wt) and (B) LeprL536Hfs*6 with mutated sequence highlighted in grey
1

## Slide 2
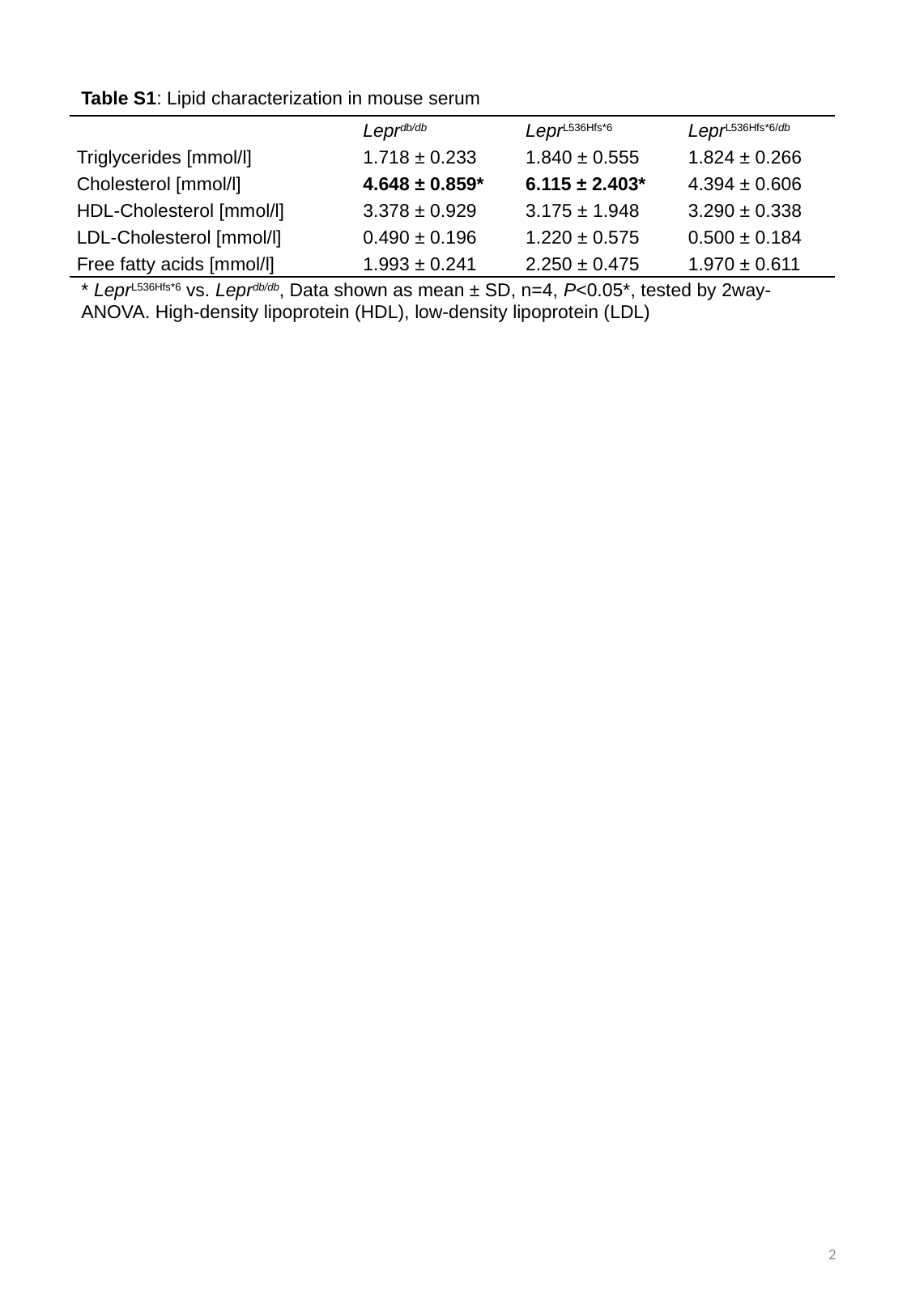

Table S1: Lipid characterization in mouse serum
* LeprL536Hfs*6 vs. Leprdb/db, Data shown as mean ± SD, n=4, P<0.05*, tested by 2way-ANOVA. High-density lipoprotein (HDL), low-density lipoprotein (LDL)
| | Leprdb/db | LeprL536Hfs\*6 | LeprL536Hfs\*6/db |
| --- | --- | --- | --- |
| Triglycerides [mmol/l] | 1.718 ± 0.233 | 1.840 ± 0.555 | 1.824 ± 0.266 |
| Cholesterol [mmol/l] | 4.648 ± 0.859\* | 6.115 ± 2.403\* | 4.394 ± 0.606 |
| HDL-Cholesterol [mmol/l] | 3.378 ± 0.929 | 3.175 ± 1.948 | 3.290 ± 0.338 |
| LDL-Cholesterol [mmol/l] | 0.490 ± 0.196 | 1.220 ± 0.575 | 0.500 ± 0.184 |
| Free fatty acids [mmol/l] | 1.993 ± 0.241 | 2.250 ± 0.475 | 1.970 ± 0.611 |
2

## Slide 3
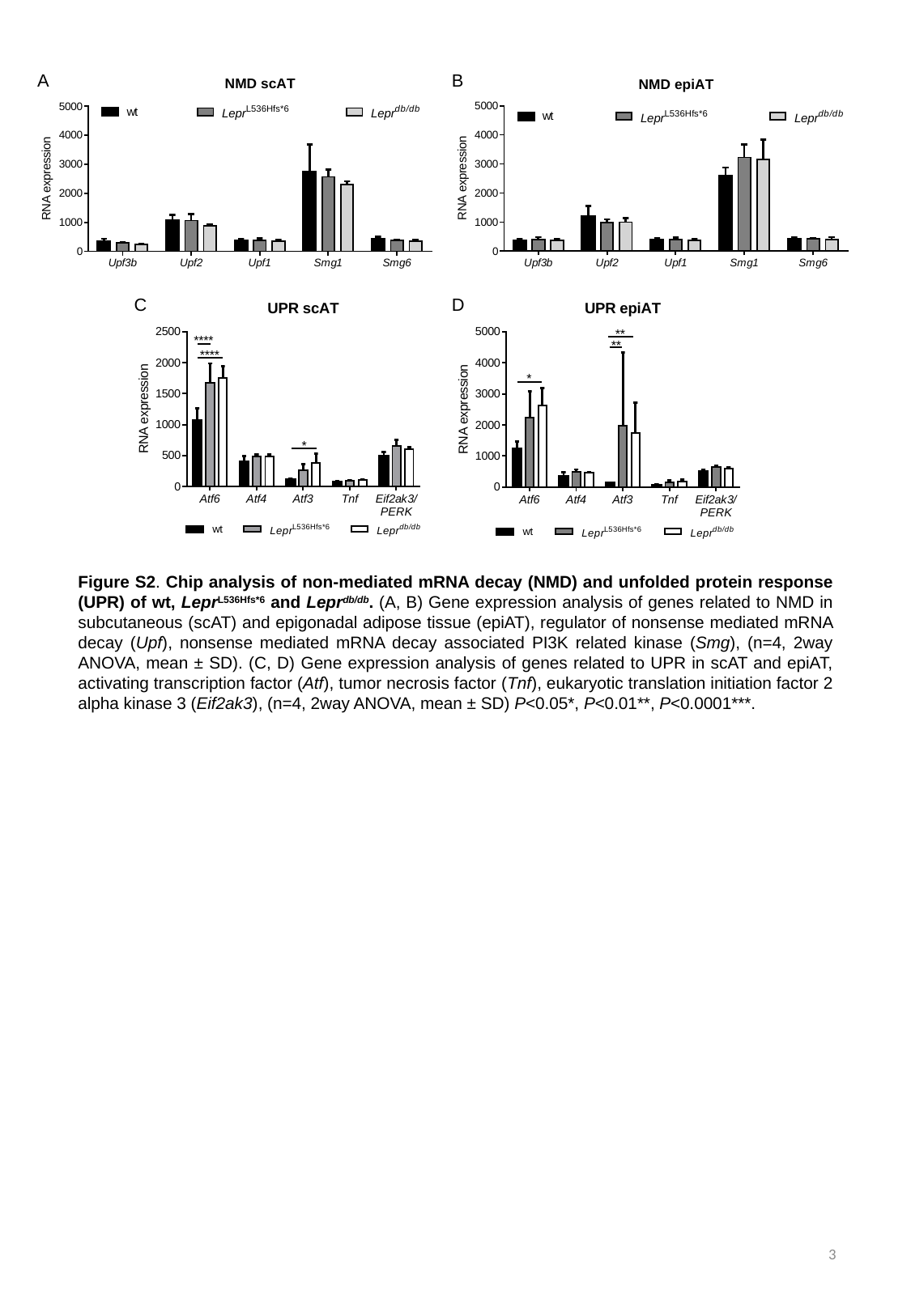

A
B
C
D
Figure S2. Chip analysis of non-mediated mRNA decay (NMD) and unfolded protein response (UPR) of wt, LeprL536Hfs*6 and Leprdb/db. (A, B) Gene expression analysis of genes related to NMD in subcutaneous (scAT) and epigonadal adipose tissue (epiAT), regulator of nonsense mediated mRNA decay (Upf), nonsense mediated mRNA decay associated PI3K related kinase (Smg), (n=4, 2way ANOVA, mean ± SD). (C, D) Gene expression analysis of genes related to UPR in scAT and epiAT, activating transcription factor (Atf), tumor necrosis factor (Tnf), eukaryotic translation initiation factor 2 alpha kinase 3 (Eif2ak3), (n=4, 2way ANOVA, mean ± SD) P<0.05*, P<0.01**, P<0.0001***.
3

## Slide 4
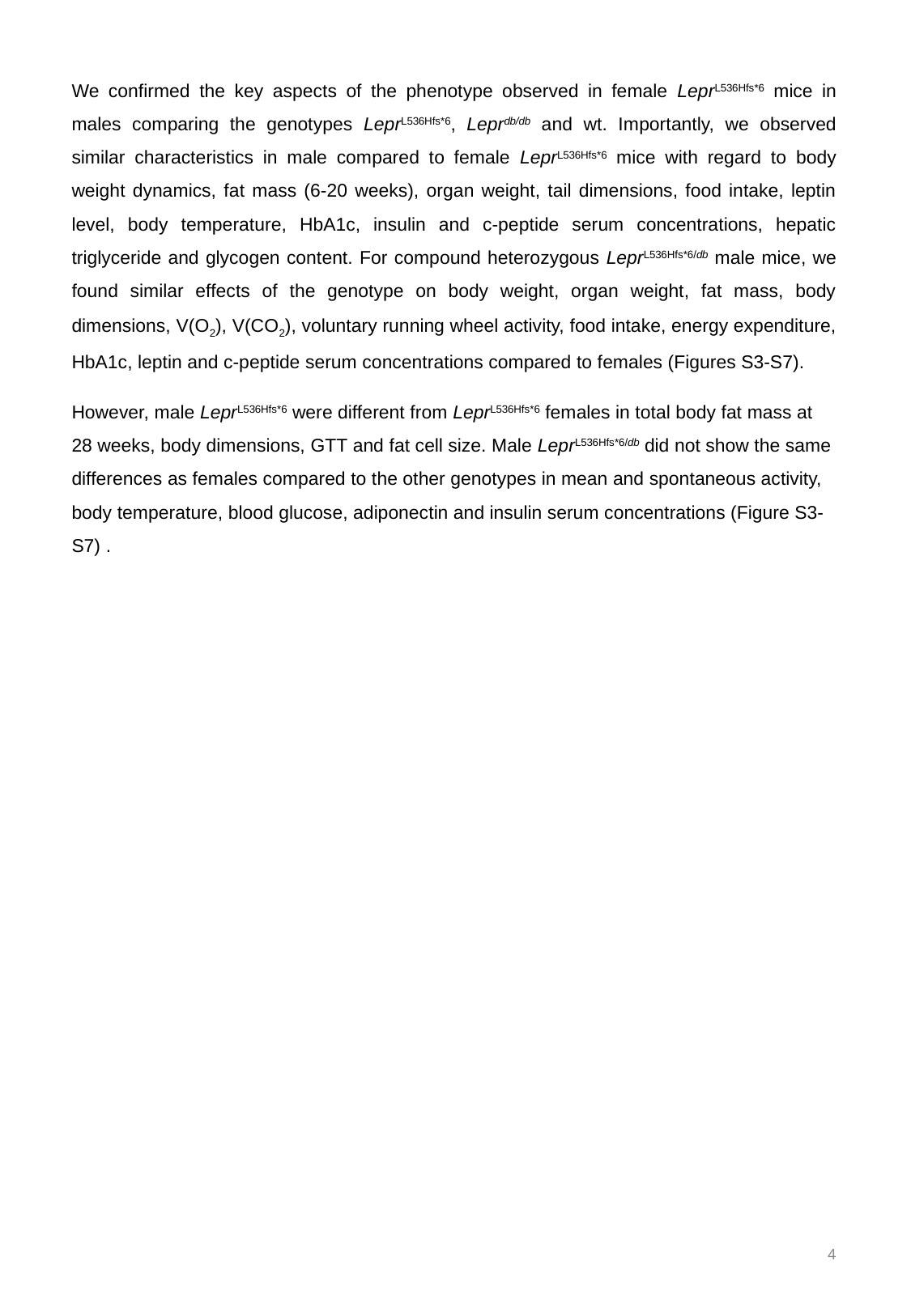

We confirmed the key aspects of the phenotype observed in female LeprL536Hfs*6 mice in males comparing the genotypes LeprL536Hfs*6, Leprdb/db and wt. Importantly, we observed similar characteristics in male compared to female LeprL536Hfs*6 mice with regard to body weight dynamics, fat mass (6-20 weeks), organ weight, tail dimensions, food intake, leptin level, body temperature, HbA1c, insulin and c-peptide serum concentrations, hepatic triglyceride and glycogen content. For compound heterozygous LeprL536Hfs*6/db male mice, we found similar effects of the genotype on body weight, organ weight, fat mass, body dimensions, V(O2), V(CO2), voluntary running wheel activity, food intake, energy expenditure, HbA1c, leptin and c-peptide serum concentrations compared to females (Figures S3-S7).
However, male LeprL536Hfs*6 were different from LeprL536Hfs*6 females in total body fat mass at 28 weeks, body dimensions, GTT and fat cell size. Male LeprL536Hfs*6/db did not show the same differences as females compared to the other genotypes in mean and spontaneous activity, body temperature, blood glucose, adiponectin and insulin serum concentrations (Figure S3-S7) .
4

## Slide 5
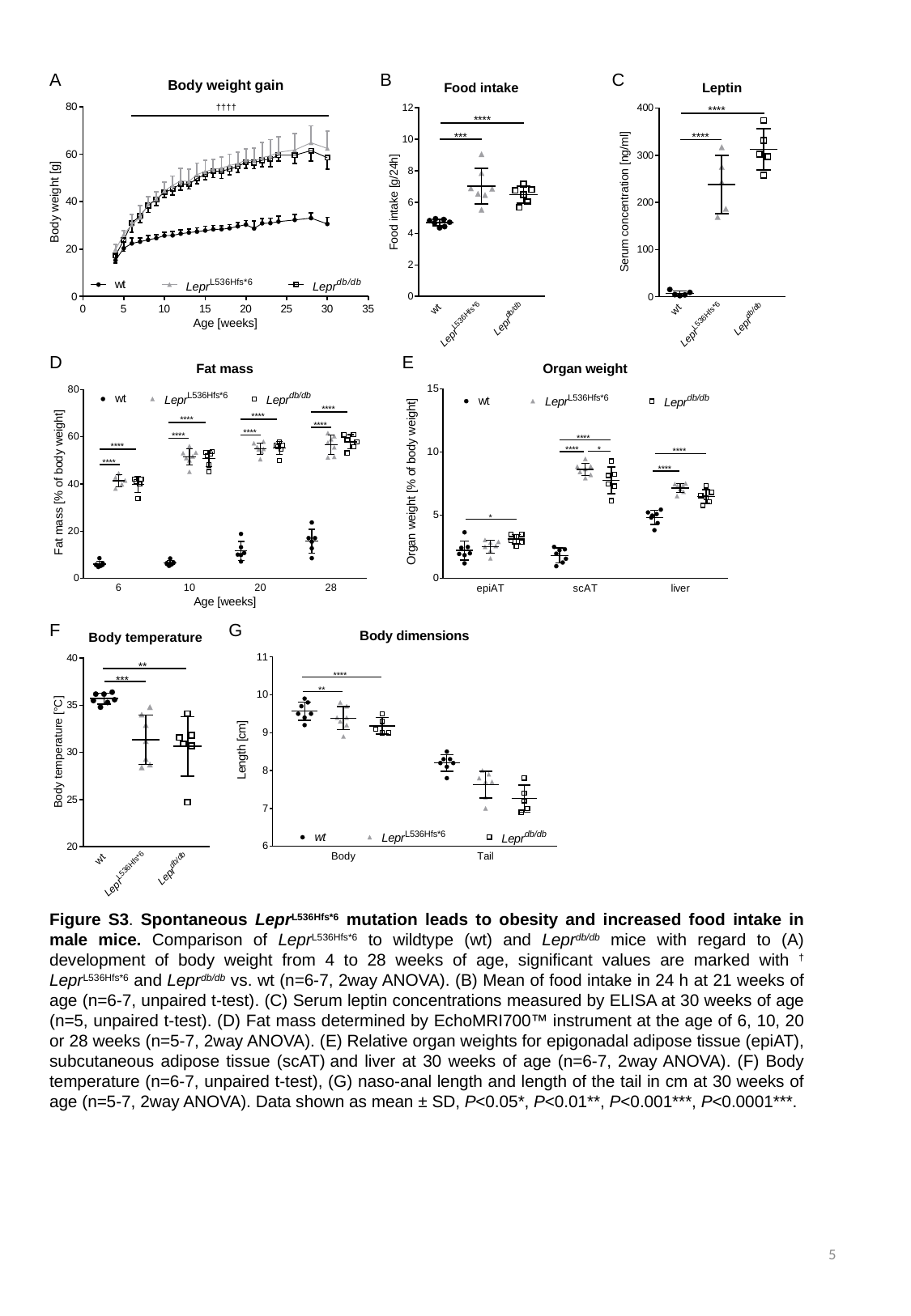

A
B
C
D
E
F
G
Figure S3. Spontaneous LeprL536Hfs*6 mutation leads to obesity and increased food intake in male mice. Comparison of LeprL536Hfs*6 to wildtype (wt) and Leprdb/db mice with regard to (A) development of body weight from 4 to 28 weeks of age, significant values are marked with † LeprL536Hfs*6 and Leprdb/db vs. wt (n=6-7, 2way ANOVA). (B) Mean of food intake in 24 h at 21 weeks of age (n=6-7, unpaired t-test). (C) Serum leptin concentrations measured by ELISA at 30 weeks of age (n=5, unpaired t-test). (D) Fat mass determined by EchoMRI700™ instrument at the age of 6, 10, 20 or 28 weeks (n=5-7, 2way ANOVA). (E) Relative organ weights for epigonadal adipose tissue (epiAT), subcutaneous adipose tissue (scAT) and liver at 30 weeks of age (n=6-7, 2way ANOVA). (F) Body temperature (n=6-7, unpaired t-test), (G) naso-anal length and length of the tail in cm at 30 weeks of age (n=5-7, 2way ANOVA). Data shown as mean ± SD, P<0.05*, P<0.01**, P<0.001***, P<0.0001***.
5

## Slide 6
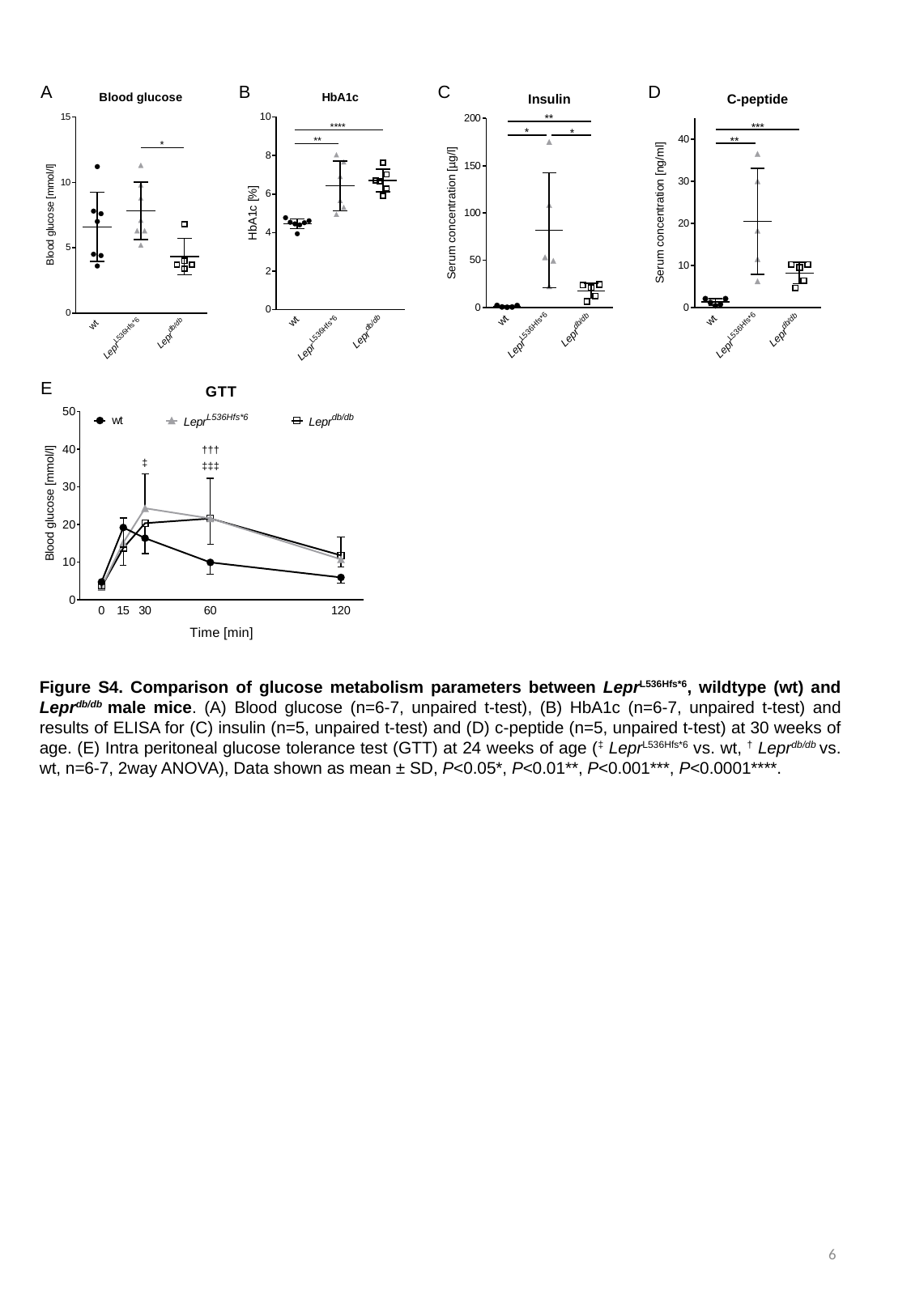

A
B
C
D
E
Figure S4. Comparison of glucose metabolism parameters between LeprL536Hfs*6, wildtype (wt) and Leprdb/db male mice. (A) Blood glucose (n=6-7, unpaired t-test), (B) HbA1c (n=6-7, unpaired t-test) and results of ELISA for (C) insulin (n=5, unpaired t-test) and (D) c-peptide (n=5, unpaired t-test) at 30 weeks of age. (E) Intra peritoneal glucose tolerance test (GTT) at 24 weeks of age (‡ LeprL536Hfs*6 vs. wt, † Leprdb/db vs. wt, n=6-7, 2way ANOVA), Data shown as mean ± SD, P<0.05*, P<0.01**, P<0.001***, P<0.0001****.
6

## Slide 7
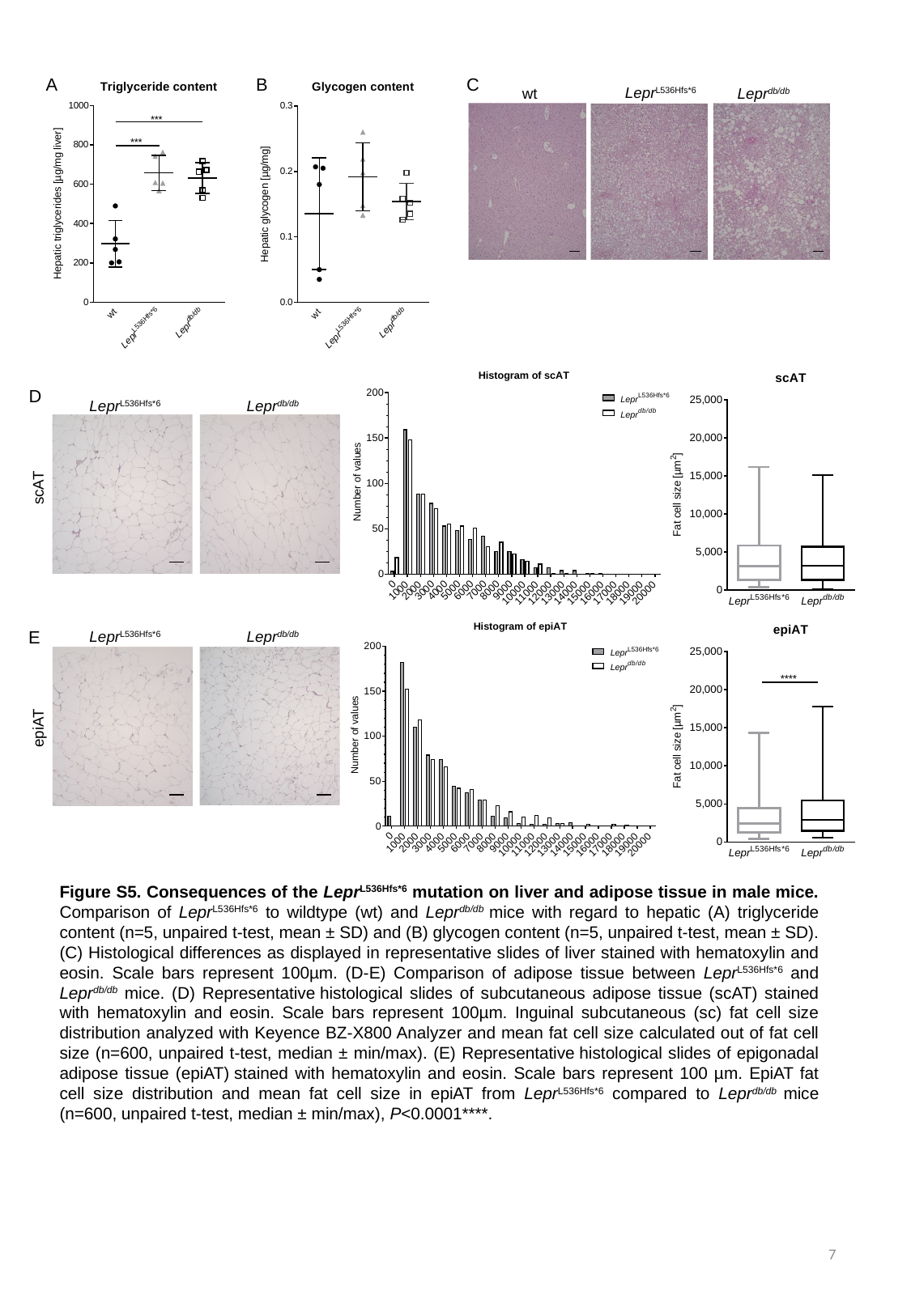

A
B
C
LeprL536Hfs*6
wt
Leprdb/db
D
LeprL536Hfs*6	 Leprdb/db
epiAT 		scAT
LeprL536Hfs*6	 Leprdb/db
E
Figure S5. Consequences of the LeprL536Hfs*6 mutation on liver and adipose tissue in male mice. Comparison of LeprL536Hfs*6 to wildtype (wt) and Leprdb/db mice with regard to hepatic (A) triglyceride content (n=5, unpaired t-test, mean ± SD) and (B) glycogen content (n=5, unpaired t-test, mean ± SD). (C) Histological differences as displayed in representative slides of liver stained with hematoxylin and eosin. Scale bars represent 100µm. (D-E) Comparison of adipose tissue between LeprL536Hfs*6 and Leprdb/db mice. (D) Representative histological slides of subcutaneous adipose tissue (scAT) stained with hematoxylin and eosin. Scale bars represent 100µm. Inguinal subcutaneous (sc) fat cell size distribution analyzed with Keyence BZ-X800 Analyzer and mean fat cell size calculated out of fat cell size (n=600, unpaired t-test, median ± min/max). (E) Representative histological slides of epigonadal adipose tissue (epiAT) stained with hematoxylin and eosin. Scale bars represent 100 µm. EpiAT fat cell size distribution and mean fat cell size in epiAT from LeprL536Hfs*6 compared to Leprdb/db mice (n=600, unpaired t-test, median ± min/max), P<0.0001****.
7

## Slide 8
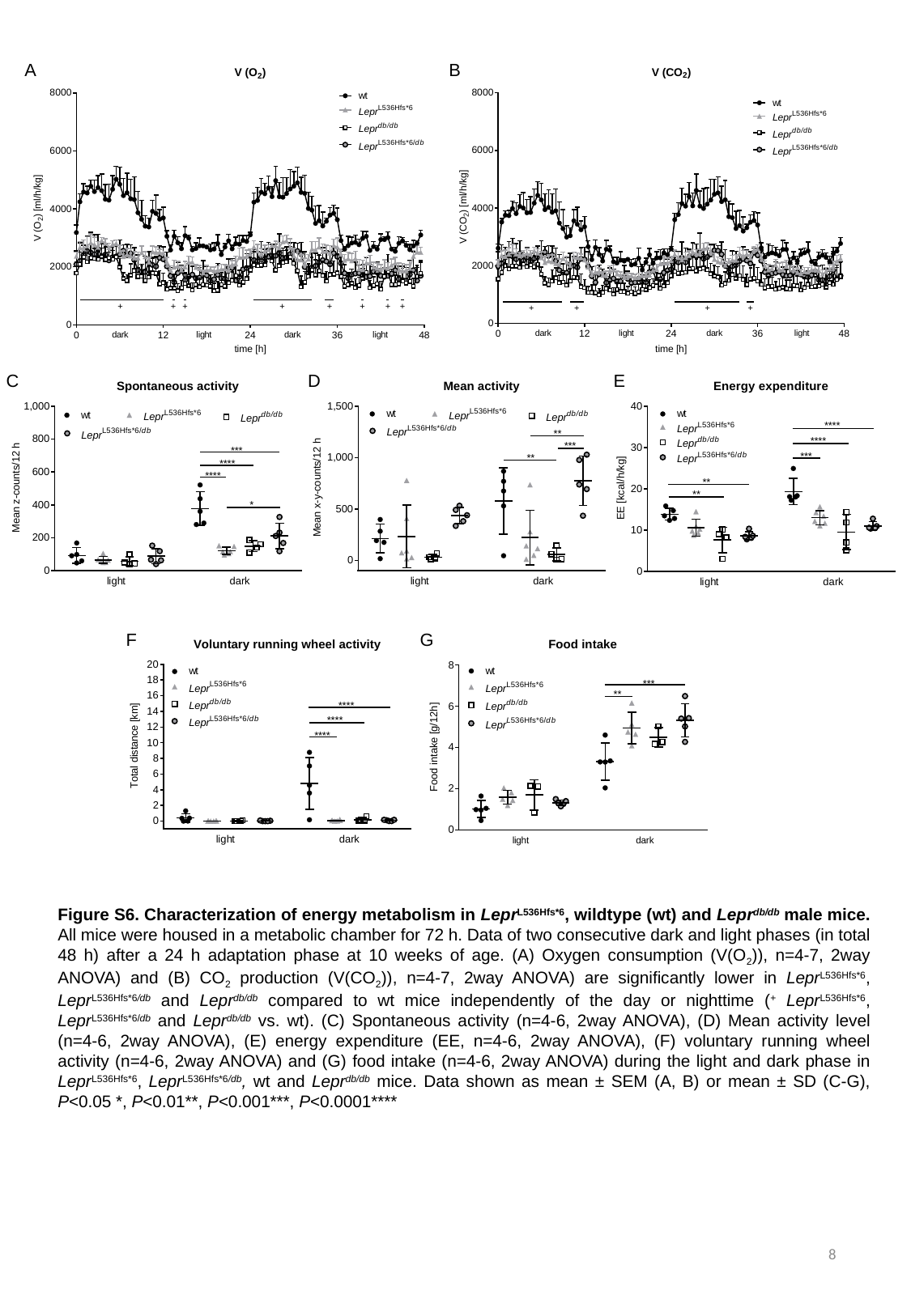

A
B
C
D
E
F
G
Figure S6. Characterization of energy metabolism in LeprL536Hfs*6, wildtype (wt) and Leprdb/db male mice. All mice were housed in a metabolic chamber for 72 h. Data of two consecutive dark and light phases (in total 48 h) after a 24 h adaptation phase at 10 weeks of age. (A) Oxygen consumption (V(O2)), n=4-7, 2way ANOVA) and (B) CO2 production (V(CO2)), n=4-7, 2way ANOVA) are significantly lower in LeprL536Hfs*6, LeprL536Hfs*6/db and Leprdb/db compared to wt mice independently of the day or nighttime (+ LeprL536Hfs*6, LeprL536Hfs*6/db and Leprdb/db vs. wt). (C) Spontaneous activity (n=4-6, 2way ANOVA), (D) Mean activity level (n=4-6, 2way ANOVA), (E) energy expenditure (EE, n=4-6, 2way ANOVA), (F) voluntary running wheel activity (n=4-6, 2way ANOVA) and (G) food intake (n=4-6, 2way ANOVA) during the light and dark phase in LeprL536Hfs*6, LeprL536Hfs*6/db, wt and Leprdb/db mice. Data shown as mean ± SEM (A, B) or mean ± SD (C-G), P<0.05 *, P<0.01**, P<0.001***, P<0.0001****
8

## Slide 9
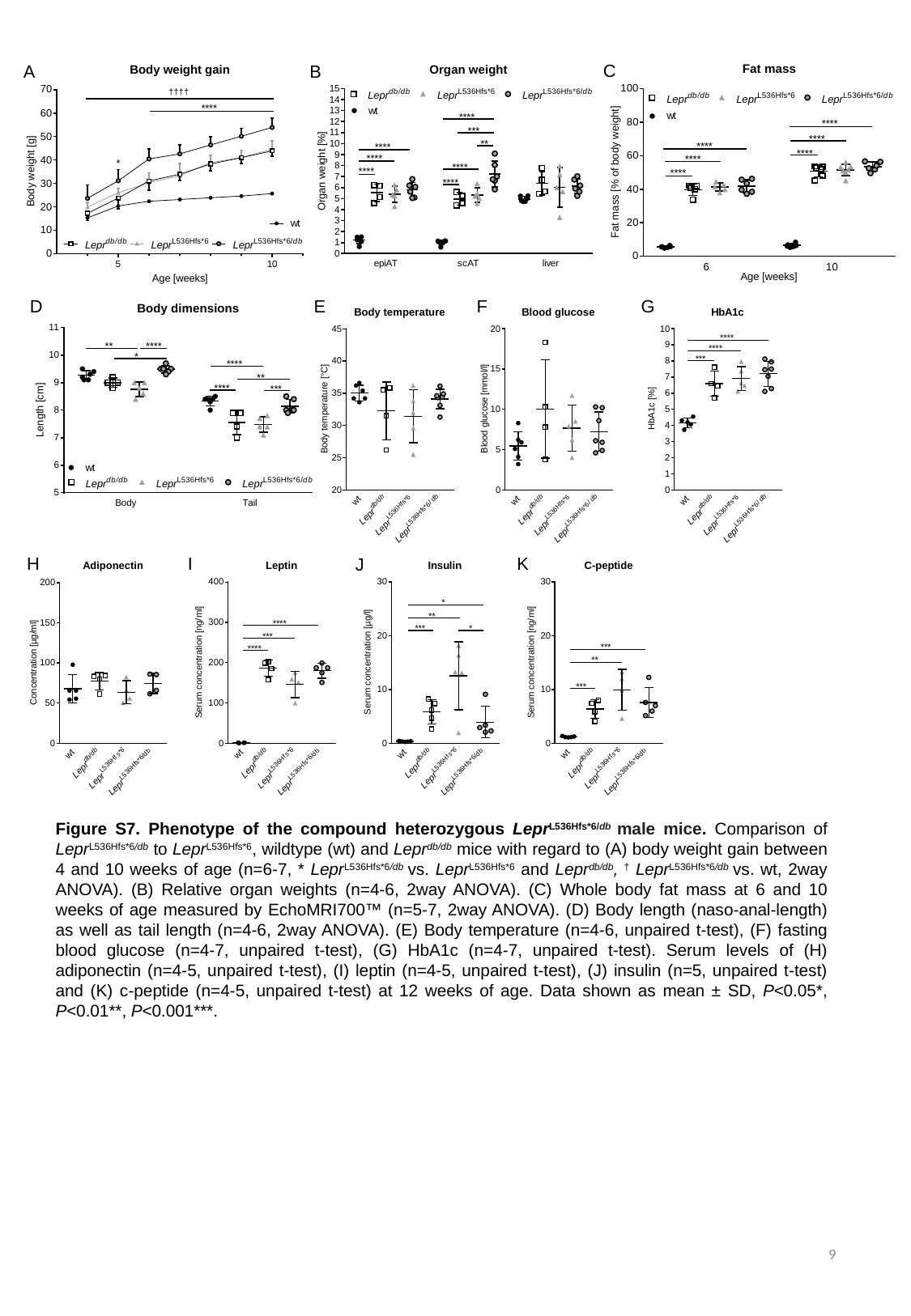

C
A
B
D
E
G
F
H
I
K
J
Figure S7. Phenotype of the compound heterozygous LeprL536Hfs*6/db male mice. Comparison of LeprL536Hfs*6/db to LeprL536Hfs*6, wildtype (wt) and Leprdb/db mice with regard to (A) body weight gain between 4 and 10 weeks of age (n=6-7, * LeprL536Hfs*6/db vs. LeprL536Hfs*6 and Leprdb/db, † LeprL536Hfs*6/db vs. wt, 2way ANOVA). (B) Relative organ weights (n=4-6, 2way ANOVA). (C) Whole body fat mass at 6 and 10 weeks of age measured by EchoMRI700™ (n=5-7, 2way ANOVA). (D) Body length (naso-anal-length) as well as tail length (n=4-6, 2way ANOVA). (E) Body temperature (n=4-6, unpaired t-test), (F) fasting blood glucose (n=4-7, unpaired t-test), (G) HbA1c (n=4-7, unpaired t-test). Serum levels of (H) adiponectin (n=4-5, unpaired t-test), (I) leptin (n=4-5, unpaired t-test), (J) insulin (n=5, unpaired t-test) and (K) c-peptide (n=4-5, unpaired t-test) at 12 weeks of age. Data shown as mean ± SD, P<0.05*, P<0.01**, P<0.001***.
9
